# Improved clinical outcome prediction in depression using neurodynamics in an emotional face-matching functional MRI task

**DOI:** 10.3389/fpsyt.2024.1255370

**Published:** 2024-03-22

**Authors:** Jesper Pilmeyer, Rolf Lamerichs, Faroeq Ramsaransing, Jacobus F. A. Jansen, Marcel Breeuwer, Svitlana Zinger

**Affiliations:** ^1^Department of Electrical Engineering, Eindhoven University of Technology, Eindhoven, Netherlands; ^2^Department of Research and Development, Epilepsy Centre Kempenhaeghe, Heeze, Netherlands; ^3^Department of Medical Image Acquisitions, Philips Research, Eindhoven, Netherlands; ^4^Department of Psychiatry, Amsterdam University Medical Center, Amsterdam, Netherlands; ^5^Department of Radiology and Nuclear Medicine, Maastricht University, Maastricht, Netherlands; ^6^Department of Biomedical Engineering, Eindhoven University of Technology, Eindhoven, Netherlands; ^7^Department of Magnetic Resonance Research & Development - Clinical Science, Philips Healthcare, Best, Netherlands

**Keywords:** major depressive disorder, prognosis, functional MRI, neurodynamics, brain networks, multi-echo

## Abstract

**Introduction:**

Approximately one in six people will experience an episode of major depressive disorder (MDD) in their lifetime. Effective treatment is hindered by subjective clinical decision-making and a lack of objective prognostic biomarkers. Functional MRI (fMRI) could provide such an objective measure but the majority of MDD studies has focused on static approaches, disregarding the rapidly changing nature of the brain. In this study, we aim to predict depression severity changes at 3 and 6 months using dynamic fMRI features.

**Methods:**

For our research, we acquired a longitudinal dataset of 32 MDD patients with fMRI scans acquired at baseline and clinical follow-ups 3 and 6 months later. Several measures were derived from an emotion face-matching fMRI dataset: activity in brain regions, static and dynamic functional connectivity between functional brain networks (FBNs) and two measures from a wavelet coherence analysis approach. All fMRI features were evaluated independently, with and without demographic and clinical parameters. Patients were divided into two classes based on changes in depression severity at both follow-ups.

**Results:**

The number of coherence clusters (nCC) between FBNs, reflecting the total number of interactions (either synchronous, anti-synchronous or causal), resulted in the highest predictive performance. The nCC-based classifier achieved 87.5% and 77.4% accuracy for the 3- and 6-months change in severity, respectively. Furthermore, regression analyses supported the potential of nCC for predicting depression severity on a continuous scale. The posterior default mode network (DMN), dorsal attention network (DAN) and two visual networks were the most important networks in the optimal nCC models. Reduced nCC was associated with a poorer depression course, suggesting deficits in sustained attention to and coping with emotion-related faces. An ensemble of classifiers with demographic, clinical and lead coherence features, a measure of dynamic causality, resulted in a 3-months clinical outcome prediction accuracy of 81.2%.

**Discussion:**

The dynamic wavelet features demonstrated high accuracy in predicting individual depression severity change. Features describing brain dynamics could enhance understanding of depression and support clinical decision-making. Further studies are required to evaluate their robustness and replicability in larger cohorts.

## 1 Introduction

Major depressive disorder (MDD) is a severe neuropsychiatric disorder and one of the leading causes of disability worldwide, affecting around 4.4% of the global population (1). Moreover, approximately one out of six people will experience a major depressive episode at some point in their lifetime (2). Yet, current treatment options are not effective: two-thirds of MDD patients will not remit after first-line treatment, which often takes nearly 3 months (3). Approximately a third develops a resistant type of depression (4). These patients often undergo various combinations of treatment strategies, including psychotherapy, multiple concurrent pharmacological treatments, switching between different antidepressants as well as changing in dose (4). Each treatment plan is based on decision-making by clinical experts, who are supported in this process by established guidelines regarding appropriate treatment and medication selection at different stages (4). However, this subjective approach might not be beneficial for all patients. It has been hypothesized that objective prognosis based on physiological measurements could support this decision-making and eventually improve the quality of individual treatment strategies (5, 6).

Functional MRI (fMRI) is an imaging technique that allows the indirect measurement of neuronal activity in the brain. Functional MR images can be acquired when the patient is at rest in the scanner (resting-state) or during the performance of a task which is developed to activate brain regions or networks associated with specific functional domains (task-based) (7). Task-based studies in MDD have demonstrated enhanced activations in limbic regions such as the amygdala and anterior cingulate cortex (ACC) in depressed subjects compared to healthy controls when viewing emotional faces (8–11). In addition, frontal activity has shown to be reduced. In terms of treatment prediction, the rostral ACC might play an important role: increased activity is often reported during processing of negative emotional faces, predicting poor treatment outcome (9, 10, 12).

Synchronicity in brain activation is another area that is often explored in fMRI research. Functional connectivity (FC), commonly measured as correlation between time-series of voxels, regions or networks, is often evaluated for this purpose. Aberrant synchronicity in depression has previously been found in both resting-state and task-based fMRI studies, showing alterations in connections between frontal, limbic, and subcortical networks (13). In addition, altered fronto-limbic FC has often been found to be significantly correlated with clinical outcome, i.e. symptomatology and treatment-induced alterations, though with inconsistent results (14, 15).

One disadvantage of FC is the fact that it is a measure of ‘static’ functional connectivity (sFC). i.e. it assumes the presence of a constant correlation and reflects the correlation between whole time-series. Yet, the activity of the brain adapts over time with complex interactions between networks. Dynamic FC (dFC) is a measure of FC that takes into account the temporal nature and interactions between time-series. Similar to sFC, it is commonly calculated by correlation, but instead over a number of shorter periods shifting over time, using a sliding window (16). This better reflects the brain activation synchronicity dynamically. For example, abnormal dFC cluster patterns, referred to as ‘brain states’, have been explored (16). Subsequently, the time of the subjects being in these states could be used for further analyses. Another common analysis approach for measuring the temporal variability is to calculate the standard deviation or dissimilarity of FC over time, reflecting the extent of synchronicity over time (17). The dFC approach has previously been successfully implemented in several resting-state MDD studies, demonstrating its potential for the purpose of objectively distinguishing between depression and controls (16, 18–21), predicting response to electroconvulsive therapy (16) or antidepressants (22) and relating to symptom severity (16, 18–20, 22, 23) and other clinical variables, such as rumination (18) and childhood trauma (23).

An alternative promising approach to explore dynamic brain activity interaction is a Wavelet Coherence Analysis (WCA). WCA yields phase information regarding two signals over time and per frequency bin: in-phase, out-of-phase, phase shifted or no coherence (24). These reflect networks that are synchronous, anti-correlated, causal or without any of these interactions, respectively. Despite its limited use in MDD research thus far, previous studies have demonstrated its potential as subjects with autism spectrum disorder could be separated from subjects without autism spectrum disorder with an accuracy of 86.7% (25). More recently, a study obtained an accuracy of 86% for classifying between depressed and non-depressed subject groups based on WCA-derived features (26).

Longitudinal approaches that aim to predict the depression course based on follow-up depression severity assessments are scarce. Moreover, the majority of MDD studies analyzed static FC in resting-state fMRI data (27, 28). Thus far, dynamic analyses of task-based fMRI have not been employed for objective depression prognosis.

Finally, fMRI acquisitions are prone to physiological and motion confounders and susceptibility artifacts in deeper located subcortical and inferior temporal regions (29–32). Multiband multi-echo (MBME) acquisitions improve the BOLD sensitivity, reduce signal loss, and allow for enhanced spatial or temporal resolution (33). The use of MBME sequences has been shown to increase sensitivity, robustness, and reproducibility in FC and dynamic co-activation pattern analyses (34, 35).

Accordingly, the goal of this study is to explore the potential of dynamic analyses on task-based MBME fMRI data for MDD prognosis. More specifically, using an emotion face-matching paradigm, we aim to predict depression symptom improvement after 3 and 6 months. Based on previous findings, we hypothesize that dynamic fMRI-based features improve the performance in predicting clinical outcome compared to activation and static FC (sFC) based features. In addition, we expect aberrant connectivity or coherence between frontal and limbic networks to be predictive of clinical outcome.

## 2 Materials and methods

**Figure 1:**
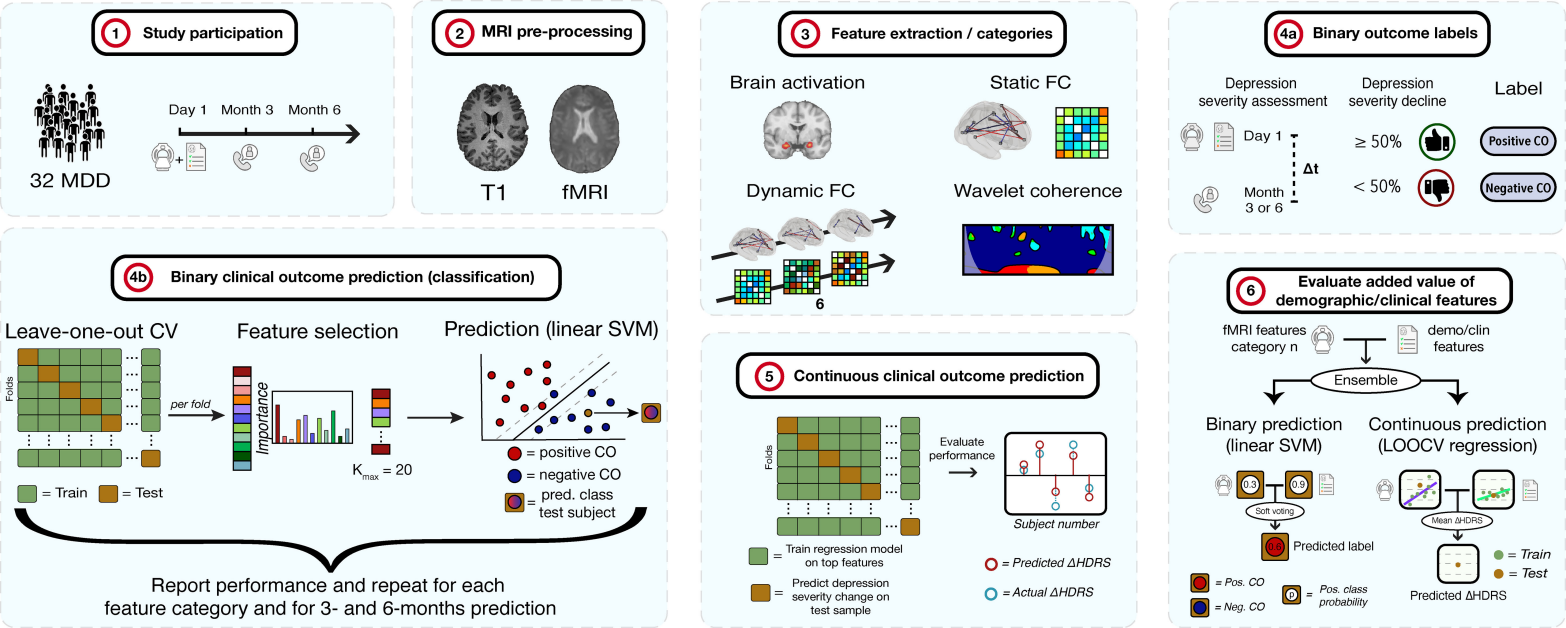
Methodology of the study. 1) 32 MDD patients are selected and complete clinical assessments and undergo an MRI examination. At 3- and 6-months, depression severity and treatment is re-assessed via telephone. 2) The T1 and fMRI scans are pre-processed. 3) fMRI features of different categories are extracted. 4a) Subjects are labeled as positive or negative clinical outcome (CO), depending on whether depression severity decreased with ≥ 50% or < 50%, respectively, after 3 or 6 months compared to baseline. 4b) For each feature category, binary CO is predicted using a linear support vector machine (SVM) classifier. Performance is assessed through leave-one-out cross-validation (LOOCV). For each fold, features are ranked and selected up to a maximum of K = 20 features. The procedure is repeated for 3- and 6-months prediction. 5) Continuous CO (change in depression severity) prediction is performed. Regression models are fit for every fold in a LOOCV procedure after which the model is applied on the test sample to obtain the predicted change in severity. These are compared to actual HDRS changes. 6) The added value of demographic and clinical features is evaluated for the binary and continuous approaches. Models of an fMRI feature set and the demo/clin features were combined by soft voting (binary) and HDRS averaging (continuous) and compared to models without the demo/clin features. HDRS, Hamilton Depression Rating Scale.

This section first describes the details of the included participants in the study. Subsequently, the study design is explained, including the clinical assessments and MRI examination. Third, the details of the MRI pre-processing steps are provided. Extraction of different categories of fMRI features are discussed in the next subsection. In the last part, the methods to predict depression clinical outcome are pointed out. These include a binary classification between the classes ≥50% versus<50% decline in depression severity, and prediction of depression severity change on a continuous scale. A summary of the methodology is shown in **Figure 1** below.

### 2.1 Participants

Thirty-two adult patients with MDD (mean age 43.8 ± 13.4 years, 20 females) satisfied selection criteria as defined hereafter and participated in the study. Patient inclusion criteria were the following: (I) age between 18 and 65 years old; (II) a diagnosis of unipolar MDD (according to the Diagnostic and Statistical Manual of Mental Disorders version 5 (36)) as assessed by board-certified psychiatrists and (III) provided written consent. Exclusion criteria included: (I) any concurrent neurological or psychiatric disorder; (II) more than 3 previous MDD episodes (excluding the current one); (III) a current MDD episode lasting longer than 2 years; (IV) previous electro-stimulation treatment; (V) current substance or alcohol abuse; (VI) a history of psychosis, autism spectrum disorder, attention deficit hyperactivity disorder or (mild) intellectual disability or (VII) any contra-indication for MRI (non-compatible brain MRI tattoos or implants, pregnancy, claustrophobia). All demographic and depression-related information can be found in **Table 1**.

**Table 1:** Demographic and clinical information of the subject group at baseline and for both, the positive and negative outcome group at 3-months and 6-months follow-up.

	Baseline	3-month follow-up			6-month follow-up		
Variable \ clinical outcome	-	Positive outcome	Negative outcome	p	Positive outcome	Negative outcome	p
***n***	32	8	24	–	12	19	–
***Age (years)***	43.8 ± 13.4	43.1 ± 13.3	44.0 ± 13.7	0.88^a^	40.3 ± 14.4	46.8 ± 12.3	0.19^a^
***Female/Male***	20/12	4/4	16/8	0.40^χ^	9/3	11/8	0.078^χ^
***HDRS score at follow-up (and at baseline)***	24.5 ± 4.59	9.88 ± 4.05 (26.9 ± 6.66)	20.1 ± 4.94 (23.7 ± 3.50)	0.00^***^ ^a^ (0.091^a^)	10.8 ± 3.36 (26.8 ± 5.83)	18.7 ± 4.66 (23.2 ± 3.17)	0.00^**^^a^ (0.034^*^^a^)
***Education level (1-5)***	1.50 ± 0.88	2.00 ± 0.76	1.33 ± 0.87	0.083^b^	1.67 ± 0.98	1.31 ± 0.75	0.25^b^
***n lifetime MDD episodes***	2.03 ± 0.69	1.88 ± 0.83	2.08 ± 0.65	0.49^b^	1.75 ± 0.62	2.26 ± 0.65	0.043^*^^b^
***Duration current episode (months)***	11.5 ± 6.33	9.25 ± 6.30	12.3 ± 6.29	0.25^a^	10.3 ± 6.69	12.1 ± 6.21	0.45^a^
***First MDD onset (years)***	33.8 ± 13.4	29.9 ± 12.5	35.0 ± 13.7	0.21^b^	32.5 ± 14.8	34.9 ± 13.1	0.56^b^

Normality of distributions was tested using a Shapiro-Wilk test. ^a^p-value calculated from a two-sample t-test (normal distribution); ^b^p-value calculated from a Mann-Whitney U test (non-normal distribution); ^χ^p-value calculated from a χ^2^ test. p-values < 0.05, 0.01 and 0.001 are indicated with *, ** and ***, respectively. Bold text indicates significant group differences. HDRS, Hamilton Depression Rating Scale; MDD, major depressive episode.

All patients gave written informed consent to participate voluntarily in this study minimally a week after being informed about the study procedures. The study was approved by the Medical Ethical Review Commission Maxima Medical Centre, Veldhoven, the Netherlands (W20.054). The clinical study is registered at clinicaltrials.gov with identifier NCT05701267.

### 2.2 Study design

This study aims to predict the change in depression severity after 3 and 6 months, based on fMRI scans of the brain at the start of the study (t=0). Therefore, at t=0 and from here on referred to as ‘baseline’, each participant underwent an MRI examination including an anatomical and a task-based functional MRI scan with an emotional faces matching paradigm. The depression severity was obtained from on-site assessments by board-certified psychiatrists at baseline. At 3- and 6-months follow-up, the depression severity was assessed again by phone to obtain the change in severity compared to baseline. One MDD patient did not complete the 6-month follow-up, leaving thirty-one patients for the 6-months analyses.

#### 2.2.1 Clinical assessments

Depression severity was measured using the 17-items Hamilton Depression Rating Scale (HDRS) (37). The total score of the items from this questionnaire reflects the depression severity, ranging from 0 to 52. Higher scores indicate more severe depression symptoms.

At baseline, participants also completed two additional questionnaires which focus more on specific domains of MDD symptoms. The state anxiety was measured with the Spielberger State-Trait Anxiety Inventory Dutch Y-form 1, containing 20 items related to the current anxiety state (38, 39). Childhood trauma was assessed using the 28-items childhood trauma questionnaire Dutch version (40, 41).

Other clinical variables that were obtained at baseline included the onset of the first depressive episode, number of lifetime episodes and duration of the current episode.

Participants received treatment as usual, i.e. therapies and medication were prescribed by their own external clinical experts (psychologists, psychiatrists) or no treatment at all. Importantly, this means that during this clinical study there was no intervention of the clinical treatment strategy.

#### 2.2.2 MRI acquisition

All participants underwent MRI scanning at Expertise center for epilepsy and sleep disorders Kempenhaeghe (Heeze, the Netherlands) using a Philips Achieva dStream 3T scanner (Philips Healthcare, Best, the Netherlands). The anatomical T1-weighted images were acquired with a 3D turbo field echo sequence with the following parameters: 1mm isotropic voxel resolution (256 x 256 x 180 matrix), repetition time (TR) = 8.1 ms, echo time (TE) = 3.7 ms, flip angle = 8°C, compressed SENSE accelerating factor = 4.6. The fMRI images were acquired using an MBME echo-planar imaging sequence with a 2.29 x 2.29 x 2.70 mm3 voxel resolution (96 x 96 x 51 matrix), 380 volumes, TR = 1350 ms, number of echoes = 3 at TE = 11.3, 31.8, 52.3 ms, flip angle = 73°C, multiband factor 3, SENSE accelerating factor 2.5. During MRI acquisition, a photoplethysmographic unit was placed on a finger and a respiratory belt was placed on the abdomen to externally measure cardiac and respiratory signals, respectively.

#### 2.2.3 Emotion processing paradigm

During the fMRI scan, participants performed an adapted version of the Hariri task: an emotion face-matching task (42). Participants were presented with blocks of either a cross (rest period), shapes or angry/fearful faces. They were instructed to match shapes or faces using a button press (left and right button). In total, there were 7 rest, 6 shapes and 6 faces blocks, lasting 27 seconds each (20 fMRI volumes). During each shapes and faces block, 4 seconds of instructions (cue) were shown, followed by 6 stimuli lasting 3 seconds each with an inter-stimulus interval of 1 second. An illustration of the task is shown in **Supplementary Figure 1**.

### 2.3 MRI preprocessing

The preprocessing was performed using Statistical Parametric Mapping software (SPM12, https://www.fil.ion.ucl.ac.uk/spm/, RRID : SCR_007037) in Matlab R2022b (The MathWorks Inc, Natick, Massachusetts, RRID : SCR_001622) and additional functions from the FMRIB Software Library v6.0 (FSL, RRID : SCR_002823) package (43). First, minimal preprocessing was performed before echo time-series combination, as described previously (44). This included the following steps:

- Slice timing correction on each separate echo time-series (SPM12)
- Realignment transformation (6 degrees-of-freedom) estimation on the echo 2 (*TE* = 31.8 ms) time-series using the first volume as reference image (FSL’s MCFLIRT function)
- Applying these estimated realignment parameters to all echo time-series (FSL’s FLIRT function)

Subsequently, the three echo time-series were combined based on the ‘optimal combination’ algorithm (45), which takes into account the varying transverse relaxation time T_2_^*^ that depends on the location of the brain (46). To combine the time-series, a weighted average was applied using weights that were calculated for each voxel according to Equation 1.


(1) $${\text{w}}_{\text{n}\;}=\frac{\text{T}{\text{E}}_{\text{n}}*{\text{e}}^{\frac{-\text{T}{\text{E}}_{\text{n}}}{{\text{T}}_{2}*}}}{\sum_{\text{i}=1}^{3}\text{T}{\text{E}}_{\text{i}}*{\text{e}}^{\frac{-\text{T}{\text{E}}_{\text{i}}}{{\text{T}}_{2}*}}},$$

Where *w_n_* = the voxel’s weight for echo *n*, *TE_n_* = echo time of echo *n* and T_2_^*^ = the voxel’s relaxation time, estimated by fitting a log function of signal decay over *TE*.

Following multi-echo combination, the T1-weighted scan was coregistered to the functional reference image using a normalized mutual information cost function. The coregistered anatomical image was then segmented into six separate classes (white and gray matter, cerebral spinal fluid, bone, soft tissue and air) based on prior tissue probabilities from a brain template in Montreal Neurological Institute standard space. During the segmentation process, linear 12 degrees-of-freedom transformation matrices for spatial normalization to Montreal Neurological Institute space were estimated. Next, spatial normalization was performed by applying these transformation matrices to the functional images. The coregistration, segmentation, and spatial normalization steps were all performed in SPM12. A conservative bandpass filter with 0.01 and 0.2 Hz cutoffs was applied to the time-series as the *TR* is relatively small (i.e. less aliasing) and artifact-like components will be removed at a later stage. This was implemented in a custom-made Matlab function using a second order zero-phase digital Butterworth filter (based on the butter and filtfilt functions). Finally, spatial smoothing with a 5 mm full-width at half-maximum kernel was applied to the functional images in SPM12.

### 2.4 Feature extraction

#### 2.4.1 Demographic and clinical features

Three demographic variables were obtained: age, sex and education (level 1-5). Clinical variables included the depression history (number of previous episodes and onset of the first episode), current depressive episode information (duration and baseline severity (HDRS) of the current episode) and questionnaires scores (regarding anxiety and childhood trauma).

Furthermore, treatment and medication information was obtained at baseline, 3-months and 6-months follow-up. Only the baseline information was used for classification as the purpose of this study is to predict future clinical outcome at the same day of scanning instead of navigating or altering treatment or medication at several intervals. Medication feature vector elements were the following: patients received any type of antidepressant treatment at baseline during the last month (yes or no); patients received selective serotonin reuptake inhibitors (SSRIs), serotonin and norepinephrine reuptake inhibitors (SNRIs), noradrenaline and specific serotonergic antidepressants (NASSAs), tricyclic antidepressants (TCAs) and/or serotonin antagonist and reuptake inhibitors (SARIs) during the last month (yes or no for each separately). Other treatment feature vector elements were the following: patients received any type of non-antidepressant treatment during the last month (yes or no); patients received cognitive behavioral therapy (CBT) (yes or no); individual (psycho)therapy (including conversations with a psychologist/psychiatrist, psychotherapy, Eye Movement Desensitization and Reprocessing, systemic therapy) or group (psycho)therapy (yes or no); paramedical profession (psychomotor or expressive therapy) (yes or no); support from a mental health institution or parental support (yes or no) during the last month.

A total of 20 elements were extracted for demographic and clinical combined feature vector.

#### 2.4.2 Brain activation

For each participant, a design matrix was assembled to obtain the activation contrast maps. First, for the physiologically-derived regressors, the externally measured cardiac and respiratory signals were used as input to the RETROICOR software tool (47). This tool models the physiological signals and derivations thereof, while taking into account the phase changing nature, yielding input regressors. Twenty-eight RETROICOR regressors were included in total: the 3 task conditions, 6 cardiac, 8 respiratory, 4 multiplicative, 1 respiration-volume-per time x respiratory response function and 6 motion regressors. By modeling the fMRI signal in a design matrix, including BOLD signals originating from the task but also the expected artifacts and noise, it is possible to estimate the explained fMRI signal contribution by the three conditions of interest (rest, shapes and faces). After modeling the design matrix, whole-brain t-value maps were calculated from the task regressors for the Faces > Shapes and Faces > Rest contrasts. Mean contrasts were calculated for the following ROIs: The bilateral amygdalae and hippocampi (from the Harvard-Oxford atlas, available within FSL https://fsl.fmrib.ox.ac.uk/fsl/fslwiki/Atlases), and the bilateral parahippocampal and fusiform gyri, as well as the ventromedial prefrontal cortex, anterior cingulate cortex (ACC) and subgenual ACC [from the automated Anatomical Labelling Atlas 3 (48)]. Previous studies have demonstrated altered activity in these regions for MDD patients compared to healthy controls during the Hariri task (49, 50) or are associated with regulating emotion by acting directly on the amygdala, the primary target brain region of the Hariri task. A total of 22 activation feature elements were extracted (11 ROIs for 2 contrasts of interest).

#### 2.4.3 Functional brain network extraction and selection

**Figure 2:**
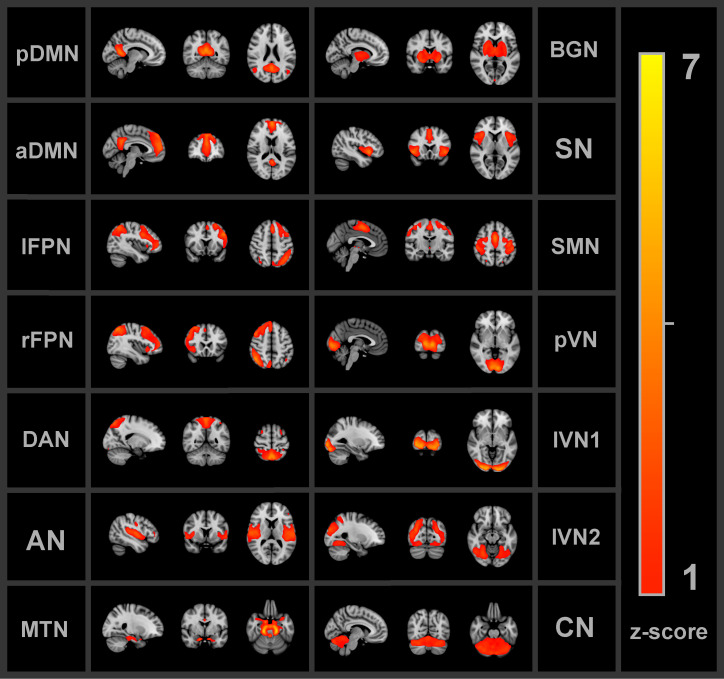
All 14 functional brain networks identified by the independent component analysis and used for the functional connectivity analyses. pDMN/aDMN, posterior/anterior default mode network; lFPN/rFPN, left/right frontoparietal network; DAN, dorsal attention network; AN, auditory network; MTN, medial temporal network; BGN, basal ganglia network; SN, salience network; SMN, sensorimotor network; pVN, primary visual network; lVN1/lVN2, lateral visual network 1/2; CN, cerebellum network.

Group independent component analysis (ICA) was applied to extract FBNs. The Group ICA of fMRI Toolbox (GIFT v3.0c, http://icatb.sourceforge.net/, RRID : SCR_001953) was implemented. First, principal component analyses reduced the data dimensions of the fMRI time-series. The Infomax algorithm (51), which maximizes the information from the input to the output of a network non-linearly, was applied to the concatenated dimensionality-reduced data, resulting in 30 spatially independent components (ICs) on a group level. The value of 30 ICs was chosen such that there was a minimum amount of subnetworks or merged networks within a single component (52). Group ICA back-reconstruction was applied to the group components to obtain the individual subject spatial maps and time-series of the ICs (53). The time-series were converted to z-scores. A goodness-of-fit approach was used between each IC and each FBN from the Smith et al. (54) FBN atlas (54) to identify the corresponding FBN. The average z-score of voxels of an extracted IC that fall outside of the FBN template was subtracted from the average z-score of voxels that fall within the FBN template (25). For each IC, the largest distance, reflecting more correspondence between the extracted IC and FBN template, was used to select the most similar FBN. Visual inspection was used to verify the automated identification process. Nine out of the ten FBNs from the atlas were identified among the ICs: the DMN, left and right frontoparietal network, auditory network, sensorimotor network (SMN), primary visual network (pVN), two lateral visual networks (lVN1 and lVN2) and the cerebellum network (CN). The DMN was separated into the anterior and posterior components (aDMN and pDMN). Additional networks that are not part of the Smith et al. atlas were found. The salience network, basal ganglia network (BGN) and DAN were identified based on previous FBN studies (55–58). Moreover, a network that comprised part of the amygdalae, hippocampi and brain stem will be referred to as the medial temporal network (MTN). Thus, in total 14 FBNs were found as shown in **Figure 2** below.

#### 2.4.4 Static and dynamic functional connectivity

**Figure 3:**
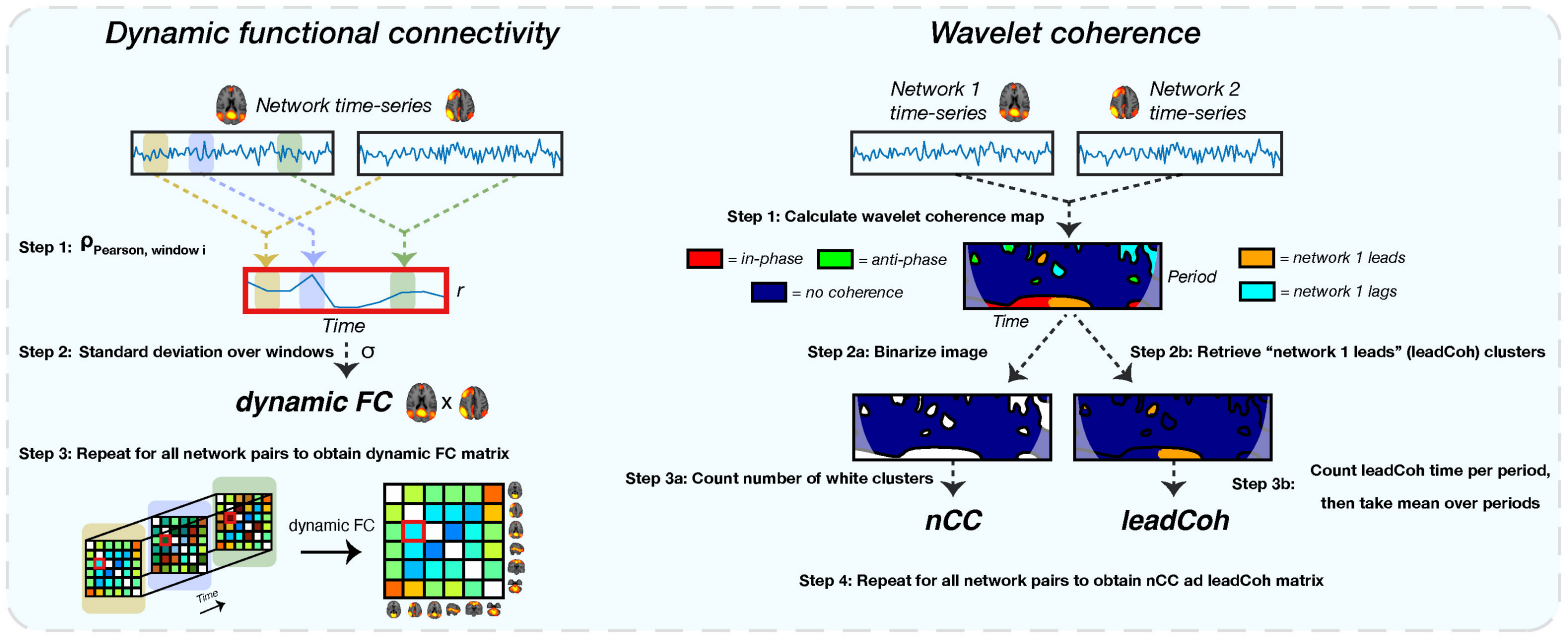
A schematic of the extraction of all dynamic functional features. The left shows the extraction of the dynamic functional connectivity (dFC) feature. For each pair of network time-series, correlations between sliding windows are obtained using Pearson correlation (step 1). This results in a correlation time-series. Subsequently, the standard deviation of this correlation time-series is obtained (step 2), representing the dFC. For the wavelet coherence feature (right side), a wavelet coherence map is obtained between each network pair (step 1). This map represents the phase relation between the two time-series for each time and period. For the number of coherence clusters (nCC), the map is binarized (step 2a): label 1 for each pixel with any type of coherence and 0 for no coherence) after which the number of clusters is counted (step 3a). Lead coherence (leadCoh) is extracted by selecting all pixels with the time-series of network 1 leading compared to network 2 (step 2b). Then, all pixels are summed over time for each period bin after which the average over bins is calculated (step 3b), representing the total average time that network 1 leads.

Static and dynamic FC was calculated between the time-series of all possible FBN pairs (n = 91), excluding identical network pairs and double pairs. For the sFC, a custom made Matlab script was implemented which calculated Pearson correlation between the time-series for the 91 pairs. The Pearson correlations were converted to z-scores. The GIFT toolbox was used to extract dFC scores between the pairs. The dFC was calculated by using a time-sliding window approach, see **Figure 3**. The window was obtained by convolution of a rectangle function of width = 30 TRs = 40.5 s [which has been found as a good balance between capturing sufficient dynamic changes and obtaining reliable dFC (59)] with a Gaussian function (σ = 3 TRs) and sliding steps of 1 TR. This yielded 350 windows in total per subject. Per FBN pair, the dFC was calculated by taking the standard deviation over the correlations between the 350 windows. A total of 91 sFC and 91 dFC feature elements were extracted (all 91 unique FBN combinations).

#### 2.4.5 Wavelet coherence analysis

Finally, a WCA was performed on the FBN pairs to assess different types of time-varying interactions between network pairs, see **Figure 3**. Here, we use the two WCA-derived features from a previous study which showed high accuracy of discriminating between depressed and non-depressed patients (26). The first feature, lead coherence (leadCoh), is an indicator of causality between FBNs. It is calculated from a WCA map by summing the time periods of a phase lead of the time-series of an FBN with respect to the time-series of another FBN and averaging over the frequency bins. The second metric, the number of coherence clusters (nCC), reflects the activation discontinuity and is calculated by counting the number of clusters in the whole WCA map of any type of coherence. Initially, the WCA map gets binarized, assigning the pixels that have any type of coherence the label 1 and all with no coherence as 0. Subsequently, a cluster is defined as a region of pixels with label 1 which have at least one adjacent pixel with label 1. The total number of clusters represents the nCC value for that pair of networks. With an increasing number of clusters, the more frequently the two networks display any type of coherence (turn on or off coherence). See Cîrstian et al. (26) for more details of how these features were extracted (26). A total of 50 periods were obtained for the analysis. For the WCA features, the bins with periods< 5 s (> 0.2 Hz) were discarded because of the previously applied bandpass filter. A total of 182 leadCoh (all 182 non-self FBN combinations; for leadCoh the matrix is not symmetrical) and 91 nCC feature elements were extracted (all 91 unique FBN combinations).

### 2.5 Clinical outcome prediction

Following feature extraction, clinical outcome was predicted. This was conducted using a binary classification and a continuous regression-based approach. For the binary method (step 4a and 4b in **Figure 1**), two clinical outcome classes were defined based on the change in depression severity (details in section 2.5.1 below). The predictive value of separating the classes was assessed independently for each of the feature categories and for the 3- and 6-months prediction using a linear support vector machine (SVM) classifier with leave-one-out cross-validation (LOOCV). Because a binary approach does not take into account subjects whose severity change is close to the cutoff value of 50%, multiple linear regression with LOOCV is implemented, predicting the HDRS change on a continuous scale, see step 5 in **Figure 1**. Finally, the potentially added value of demographic and clinical information to the fMRI features was evaluated using an ensemble of binary and continuous classifiers, see step 6 in **Figure 1**. For each ensemble of two binary linear SVM classifiers, soft-voting was implemented, averaging the class probability from both classifiers. For each ensemble of two regression models, the average predicted HDRS change from both classifiers was calculated.

#### 2.5.1 Binary classification

For the binary classifications, participants were labeled into two classes with different clinical outcomes. First, changes in the total HDRS score (reflecting depression severity) with respect to total baseline HDRS score were calculated according to Equation 2.


(2) $$\text{Δ}\;\text{HDRS}=\;\frac{\text{HDR}{\text{S}}_{\text{FU}}-\;\text{HDR}{\text{S}}_{\text{baseline}}}{\text{HDR}{\text{S}}_{\text{baseline}}}*100\%$$

With *HDRS_FU_* = total HDRS-17 score at either the 3- or 6-months follow-up and *HDRS_baseline_* = total HDRS-17 score at baseline. Then, participants with a *ΔHDRS* ≤ 50% were labeled as ‘positive outcome’ and the others as ‘negative outcome’ as this cutoff has been accepted and validated as the golden standard for evaluation of clinically significant improvement (60, 61). The binary classification was done separately for the 3-months and 6-months predictions. After 3-months, n = 8 versus n = 24 subjects were labeled as positive and negative outcome, respectively. After 6-months, n = 12 versus n = 19 subjects were labeled as positive and negative outcome, respectively. To assess the robustness of our findings, we also analyze the results of the predictive value of each of the feature categories when using the absolute change in HDRS as dependent variable.

SVM classifiers with a linear kernel were trained on vectors of different feature categories, separately, see **Table 2** below. Validation was performed by LOOCV for which one subject is left out for testing in each fold. The other (N-1) subjects are used to train the SVM model. For classification, a higher penalty was applied for incorrectly classifying the minority class by incorporating a cost function that was proportional to the ratio between the sample sizes of the two classes.

**Table 2:** All features that were used for clinical outcome prediction.

SVM model	Feature category	Abbreviation	Vector elements + description	Number of elements	Max K features after feature reduction
***1***	Demographics + clinical	Demo+clin	Age, sex, education, baseline questionnaire scores (k = 2), depression history (k = 2), current depressive episode information (k =2) and treatment/medication variables (k = 11)	20	20
***2***	Activity contrast	Act	Mean t-values for 11 ROIs and 2 contrasts	22	20
***3***	Static FC	sFC	sFC for all unique pairs of 14 FBNs	91	20
***4***	Dynamic FC	dFC	dFC for all unique pairs of 14 FBNs	91	20
***5***	Lead coherence	leadCoh	leadCoh for all non-self pairs of 14 FBNs (matrix values are not symmetrical for leadCoh)	182	20
***6***	Number of coherence clusters	nCC	nCC for all unique pairs of 14 FBNs	91	20

All different fMRI feature categories were used to classify clinical outcome in separate prediction models. Before prediction, each feature set was used as input to the feature reduction approach to select a maximum of K= 20 features. FC, functional connectivity; nCC, number of coherence clusters; ROIs, regions-of-interest; FBNs, functional brain networks; sFC, static FC; dFC, dynamic FC.

Because the number of components is higher (range 20 – 182) compared to the number of subjects (n = 32) for most feature categories, overfitting could occur, thereby significantly reducing the generalization of the prediction model. In order to alleviate this problem, strict feature selection was performed. For each feature category, a number of K feature elements, ranging from 1 to 20 elements, was implemented. A maximum number of K = 20 elements was chosen as the clinical and demographic feature vector contained the least number of elements of all feature categories and fair comparison between the different categories is required (62). For each round of LOOCV, K was fixed while the feature elements differed within each fold. For clarity, the first LOOCV procedure was run with K = 1. For each fold, the top K = 1 element was selected based on feature ranking of the training set of that fold. After LOOCV with K = 1, a new LOOCV procedure with K = 2 was performed. Again, the top K = 2 elements was selected for each fold. This was repeated until K = 20. Accuracy, sensitivity, specificity, precision, F1-score and area under the curve (AUC) performance metrics were obtained. The optimal K was defined as K at which LOOCV reached the highest accuracy. The total occurrence of each feature element over all folds was counted. For feature ranking and selection, two algorithms were implemented:

1. Linear SVM recursive feature elimination (SVM-RFE): An SVM-RFE fits linear SVM models on all elements in the feature vector and iteratively drops the least class-separable element. It has been previously been found to show high performance with a linear SVM in MDD diagnosis (63).
2. Kruskal-Wallis test: The Kruskal-Wallis test is a non-parametric procedure that compares the medians of feature elements between two or more groups to determine whether the samples come from the same population distribution. It does not assume normal distributions. It has been found to be an effective feature selection method in combination with SVM classifiers (64).

#### 2.5.2 Continuous clinical outcome prediction

For the prediction of HDRS severity change on a continuous scale, again, an LOOCV procedure was implemented. For each feature category and follow-up, a multiple linear regression model was fit on the data of all but one subject, i.e. training set. The values of the feature vector of the test subject were then used as input to the model to obtain the predicted change in HDRS for the left-out subject as in Equation 3:


(3) $$\Delta \text{HDR}{\text{S}}_{\text{FU}}\;∼\;{\text{x}}_{\text{c},1}*{\text{β}}_{\text{c},1}+\;{\text{x}}_{\text{c},\text{k}}*{\text{β}}_{\text{c},\text{k}}\ldots +\;{\text{x}}_{\text{c},\text{K}}*{\text{β}}_{\text{c},\text{K}},$$

with *ΔHDRS_FU_* the relative percentage change in HDRS score after 3 or 6 months as dependent variable and the top *K* = 20 elements (index *k*) of each feature category (index c) *x_c,k_* as independent variables, together with their corresponding beta-coefficient *β_c,k_*. This process was repeated for each fold. Similar as the binary classifications, feature selection was applied within each fold. Multiple LOOCV procedures were run, each time changing the number of feature elements *k*. *k* was kept constant for each fold within an LOOCV procedure. Twenty LOOCV procedures were run for *k* = 1 to *K* = 20. The performance with an optimal number of elements was reported. For each test subject, the predicted HDRS change was compared to the actual HDRS change. RMSE, mean absolute error and correlation between the predicted and actual HDRS change were reported. The correlation was tested for significance and the corresponding p-values were corrected with the Holm-Bonferroni procedure (65).

#### 2.5.3 Statistical analysis

A statistical analysis was performed to evaluate the extent to which the feature elements of the top models of the binary classification relate to HDRS change on a continuous scale in general. Note, however, that this is different from prediction since there is no unseen data provided to the model. Moreover, the fitted models over the data of all subjects give more insights in the impact and direction of change that each feature element has on a change in HDRS, i.e. how much an increase or decrease in a predictor changes the HDRS change.

For this purpose, a multiple linear regression model was fit through the data of all subjects for each feature category and follow-up, separately. The dependent variable was the HDRS severity change and the predictor variables were the top features of each feature category. First a base model was defined based on the top *K* = 20 features found for the binary classification as in Equation 3. Then, feature element elimination was performed one-by-one based on the significance of contribution to the model. That is, the feature element with the highest p-value was removed and this was repeated until the last remaining element. Performance of the most optimal model was reported. The F-statistic of the model was reported, as well as the goodness-of-fit metrics root mean square error (RMSE) and the adjusted coefficient of determination (Adj. R^2^).

#### 2.5.4 Evaluating the added value of demographic and clinical parameters

In addition to separately evaluating the fMRI-based and demo+clin features, we created ensembles of prediction models that combined both. In this way, the added value of demo+clin information to the fMRI features was assessed.

For the binary classification, ensemble models were established by combining two linear SVM models, of which one was fixed: the demo+clin feature set. The other model varied and comprised one fMRI feature category. All five possible ensemble combinations of the demo+clin and each fMRI feature category were evaluated. From each of the two linear SVM models, the positive CO class probability was obtained and averaged (soft-voting) to obtain the ensemble class probability for the positive CO class. If this class probability ≥ 0.5, the test sample was labeled as positive CO and as negative CO if it was< 0.5. Similar performance metrics as the binary prediction were calculated.

For the continuous LOOCV-based HDRS change prediction, ensemble models of two regression models were created. Again, in each ensemble model one of the models was the fixed demo+clin feature set while the other was one of the fMRI-based models. The predicted change in severity of the ensemble model was calculated as the average predicted severity change by the two models. All 5 possible combinations were tested.

## 3 Results

### 3.1 Classification-based (binary) clinical outcome prediction

**Figure 4:**
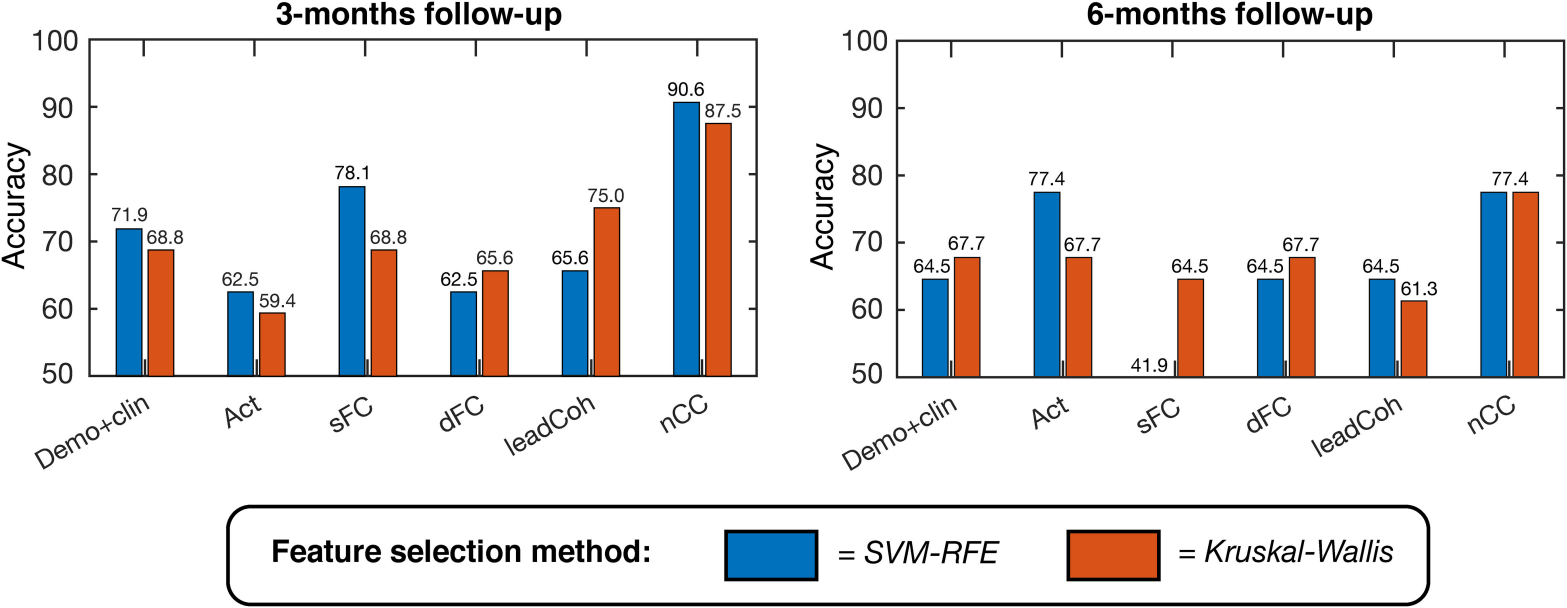
Linear support vector machine (SVM) binary classification accuracies based on leave-one-out cross-validation for predicting positive versus negative clinical outcome at 3-months (left) and 6-months (right). Several feature categories were tested, with a maximum of K = 20 features, ranging from demographic and clinical information to brain activity, static functional connectivity (sFC) and dynamic features. Two feature selection methods were used, indicated by the two colors: SVM – Recursive Feature Elimination (SVM-RFE, blue) and Kruskal-Wallis (red). demo, demographic; clin, clinical; act, activity; s/dFC, static/dynamic functional connectivity; leadCoh, lead coherence; nCC, number of coherence clusters.

For the binary outcome prediction, the classifiers based on the nCC feature category outperformed the other demo+clin and fMRI-based classifiers. A maximum accuracy of 90.6% (area under the curve (AUC) 0.813, F1-score 0.769) and 77.4% (AUC 0.770, F1-score 0.720) was obtained, respectively, see **Figure 4** and **Supplementary Table 1**. In general, SVM-RFE feature selection showed slightly higher accuracies but similar distributions between the feature categories. Nonetheless, SVM-RFE appeared to predict more subjects as negative clinical outcome than Kruskal-Wallis, as reflected by the unbalanced sensitivity-specificity scores. These scores were more balanced for the Kruskal-Wallis feature selection, which still achieved a maximum of 87.5% (for nCC) and 83.9% (for ensemble of demo+clin, s/dFC and nCC) accuracy for the 3- and 6-months predictions, respectively.

### 3.2 Continuous clinical outcome prediction

**Figure 5:**
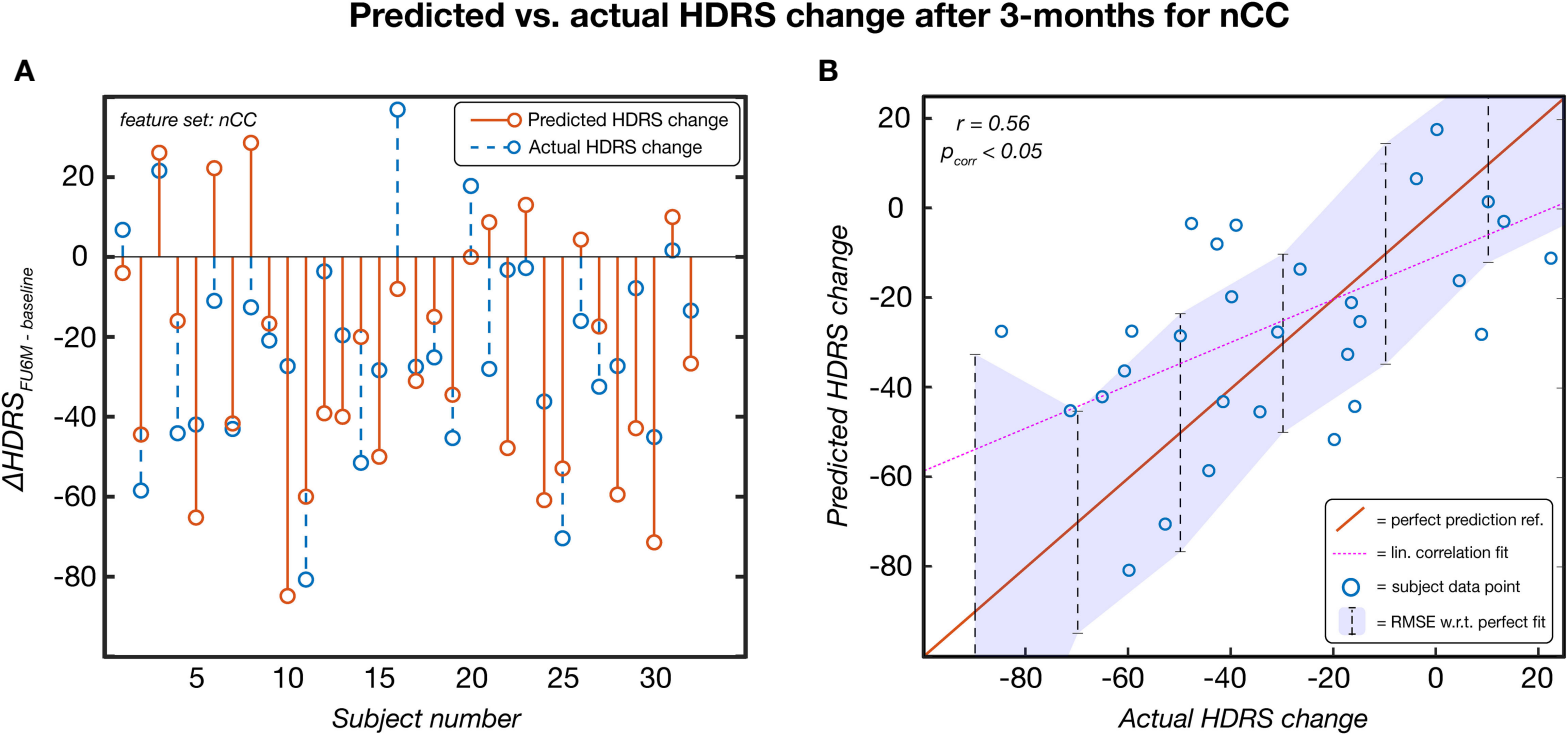
The predicted HDRS change versus the actual HDRS change after 3 months for the feature set number of coherence clusters (nCC). Predicted scores were obtained via a leave-one-out procedure. This model was established based on Kruskal-Wallis feature selection and stayed significant following multiple comparison correction (p< 0.05). **(A)** Per-subject scores of the predicted (red) and actual (blue) HDRS changes. **(B)** pairs of actual-predicted scores (blue dots) plotted on top of a perfect prediction as reference (red line). The dotted purple line indicates the linear correlation fit (r = 0.56) and the purple area indicates the root mean squared error (RMSE) of the data points with respect (w.r.t.) to the perfect fit. ref, reference; lin, linear; HDRS, Hamilton Depression Rating Scale; FU3M, 3-months follow-up.

Leave-one-out based HDRS change prediction was performed for the linear regression models of each feature category models. For each fold, the predicted severity change on the left-out test subject was compared to the actual severity change. Only the results obtained by the Kruskal-Wallis feature selection are reported here because of higher and more balanced performances. The SVM-RFE results can be found in **Supplementary Table 2**. As can be seen in **Table 3**, nCC outperformed the other models, demonstrating the highest correlation between predicted and actual severity changes for the 3-months (*r* = 0.561, p_corr_< 0.001, see **Figure 5**) and for the 6-months (*r* = 0.383, p_uncorr_< 0.05) follow-up. Of note is the low performance of leadCoh, which previously demonstrated to be one of the highest performing feature categories. Potentially, the leadCoh models were unstable, showing less individual predictive power, as the effect of removing a single subject for feature selection and model fitting significantly altered performance.

**Table 3:** Comparison between predicted and actual depression severity changes for the multiple linear regression with leave-one-out cross-validation approach.

	3-months follow-up				
Feature category	Correlation	RMSE	MAE	Optimal k	p-value (uncorr.)
***Demo/clin***	0.489	26.9	**21.9**	3	< 0.01
***Act***	0.375	27.8	22.7	1	< 0.05
***sFC***	0.065	47.9	36.1	10	0.722
***dFC***	0.071	30.9	25.2	1	0.701
***leadCoh***	-0.127	45.4	39.1	5	–
***nCC***	**0.561**	**26.4**	22.7	5	< 0.001 ^*^
	6-months follow-up				
Feature category	Correlation	RMSE	MAE	Optimal k	p-value (uncorr.)
***Demo/clin***	0.293	38.2	30.0	20	0.110
***Act***	0.330	**26.5**	**21.9**	1	0.0696
***sFC***	0.075	45.3	37.4	10	0.688
***dFC***	-0.511	35.3	29.3	1	-
***leadCoh***	-0.199	58.4	41.1	7	–
***nCC***	**0.383**	30.1	23.7	5	< 0.05

Models were fit on the N-1 training set, which was then used to predict depression severity change after 3 and 6 months of the test subject. This procedure was repeated for all subjects. Correlation, RMSE and MAE indicate the performance between predicted and actual changes in severity. The p-value corresponds to the correlation between both. Bold p-values marked with ^*^ are significant after multiple comparison correction at p< 0.05. The optimal k features elements of each category were determined by initial Kruskal-Wallis selection (K = 20) and subsequently removing the lowest significant predictors iteratively until maximum performance was reached. demo, demographic, clin, clinical; Act, activity; nCC, number of coherence clusters; s/dFC, static/dynamic functional connectivity; leadCoh, lead coherence; RMSE, root mean square error; MAE, mean absolute error.

### 3.3 Statistical analysis

When the regression models were fit over the data of all subjects, the two features categories derived from the WCA obtained the highest goodness-of-fit metrics for both follow-ups. More specifically, nCC models reached the highest performance (lowest RMSE and adjusted R^2^) for both follow-ups, followed by leadCoh, see **Table 4**. Interestingly, demo+clin features showed higher performance compared to Act, sFC and dFC models. The models for the SVM-RFE feature ranking can be found in **Supplementary Table 3**.

**Table 4:** Goodness-of-fit statistics for the optimal multiple linear regression models for each of the feature categories relating to the change in 3- and 6-month depression severity.

	3-months follow-up				6-months follow-up			
Feature category	F-statistic	RMSE	Adj. R^2^	Optimal k	F-statistic	RMSE	Adj. R^2^	Optimal k
***Demo/clin***	7.74	23.6	0.395	3	4.38	20.0	0.474	8
***Act***	4.31	24.5	0.348	5	**13.3**	23.3	0.291	1
***sFC***	6.38	26.1	0.258	2	3.98	26.3	0.0903	1
***dFC***	2.83	28.0	0.151	3	2.37	27.0	0.0438	1
***leadCoh***	**9.25**	22.6	0.444	3	8.67	18.3	0.561	5
***nCC***	7.78	**21.0**	**0.522**	5	7.23	**16.9**	**0.624**	8

The models were fit over all subjects and the optimal k features elements were determined by initial Kruskal-Wallis feature selection (K = 20) and by subsequently removing the lowest significant predictors iteratively until maximum performance was reached. Bold text indicates the highest obtained performance per column. demo, demographic, clin, clinical; Act, activity; nCC, number of coherence clusters; s/dFC, static/dynamic functional connectivity; leadCoh, lead coherence; RMSE, root mean square error; Adj. R^2^, adjusted coefficient of determination.

Furthermore, in the subsection ‘Feature analysis’ the predictors and corresponding β-coefficients of several feature categories are discussed to elucidate the directions and significance of change in the regressors.

### 3.4 Evaluating the added value of demographic and clinical parameters

The performance metrics of ensembles of binary classifiers with an accuracy > 75% can be found in **Table 5** and those of all ensembles in **Supplementary Tables 4**, **5**. Ensembles classifiers with Kruskal-Wallis feature ranking increased the accuracy for each fMRI feature category at 3-months follow-up. The most notable improvement was the ensemble of the demo+clin feature set with the leadCoh feature set. Whereas a single linear SVM predicted clinical outcome with 75.0% based on leadCoh features, the combination with the demo+clin features improved it to 81.2%. For the 6-month predictions, none of the ensembles improved the accuracy compared to a single SVM classifier. However, the ensembles with the nCC classifier still reached the highest accuracy compared to the other fMRI feature categories (except for 6-months prediction with SVM-RFE feature ranking), indicating its significant contribution.

**Table 5:** Performance of the top (> 75%) ensemble classifiers for the binary 3- and 6-month clinical outcome prediction with SVM-RFE (support vector machine – recursive feature elimination) or Kruskal-Wallis future ranking.

	3-months follow-up						
Ensemble	Feature selection	Acc	Sens	Spec	Prec	F1-score	AUC
***Demo/clin + nCC***	SVM-RFE	78.1	25.0	**95.8**	0.667	0.364	0.604
***Demo/clin + sFC***	Kruskal-Wallis	78.1	50.0	87.5	0.571	0.533	0.688
***Demo/clin + leadCoh***	Kruskal-Wallis	81.2	**62.5**	87.5	0.625	0.625	0.750
***Demo/clin + nCC***	Kruskal-Wallis	**87.5**	**62.5**	**95.8**	**0.833**	**0.714**	**0.792**
	6-months follow-up						
Ensemble	Feature selection	Acc	Sens	Spec	Prec	F1-score	AUC
***None reached Acc > 75%***	–	–	–	–	–	–	–

Bold text indicates the highest obtained performance per column. demo, demographic, clin, clinical; nCC, number of coherence clusters; sFC, static functional connectivity; leadCoh, lead coherence; Acc, accuracy; Sens, Sensitivity; Spec, specificity; Prec, precision; AUC, area under the curve.

To evaluate the effect of adding demographic and clinical variables for prediction on a continuous scale, predicted depression severity change scores were averaged over one of each of the fMRI-based models and the demo+clin model. This merely improved the prediction for sFC whereas the effect on other feature categories was minimal or even led to a decrease, see **Table 6**. Nonetheless, the performance of nCC was still the most optimal of all models. The regression prediction models based on the SVM-RFE feature ranking can be found in **Supplementary Table 6**.

**Table 6:** Linear regression performance of ensemble models between fMRI and demographic/clinical features using Kruskall-Wallis feature selection.

	3-months follow-up			
Ensemble	Correlation	RMSE	MAE	p-value (uncorr.)
***Demo/clin + Act***	0.183	42.8	33.2	0.317
***Demo/clin + sFC***	0.239	38.8	29.1	0.188
***Demo/clin + dFC***	-8.86 • 10^-3^	47.7	37.2	*-*
***Demo/clin + leadCoh***	-7.21 • 10^-3^	46.4	37.5	*-*
***Demo/clin + nCC***	**0.403**	**33.4**	**27.4**	< 0.05
	6-months follow-up			
Ensemble	Correlation	RMSE	MAE	p-value (uncorr.)
***Demo/clin + Act***	0.183	42.9	31.5	0.326
***Demo/clin + sFC***	0.199	**33.0**	28.4	0.284
***Demo/clin + dFC***	-0.343	45.4	37.3	-
***Demo/clin + leadCoh***	-0.121	45.6	33.9	-
***Demo/clin + nCC***	**0.252**	34.0	**26.4**	0.172

The metrics in this table are a comparison between predicted and actual depression severity changes using a leave-one-out cross-validation approach. Models were fit on the N-1 training set, which was then used to predict depression severity change after 3 and 6 months of the test subject. This procedure was repeated for all subjects. Correlation, RMSE and MAE indicate the performance between predicted and actual changes in severity. The p-value corresponds to the correlation between both. The ensemble predicted severity change was calculated as average between the separate demo/clin model and the separate fMRI-based models. Bold text indicates the highest obtained performance per column. demo, demographic, clin, clinical; Act, activity; nCC, number of coherence clusters; s/dFC, static/dynamic functional connectivity; leadCoh, lead coherence; RMSE, root mean square error; MAE, mean absolute error.

### 3.5 Feature analysis

To assess which parameters contributed most to the highest prediction models in general, the feature elements importance was estimated for the demo+clin features and the highest performing feature nCC. For the demo+clin category, the total number of occurrences of each parameter in the optimal models for the binary and both regression approaches was acquired. For the nCC feature, the count of each network in all the analyses was reported.

**Figure 6:**
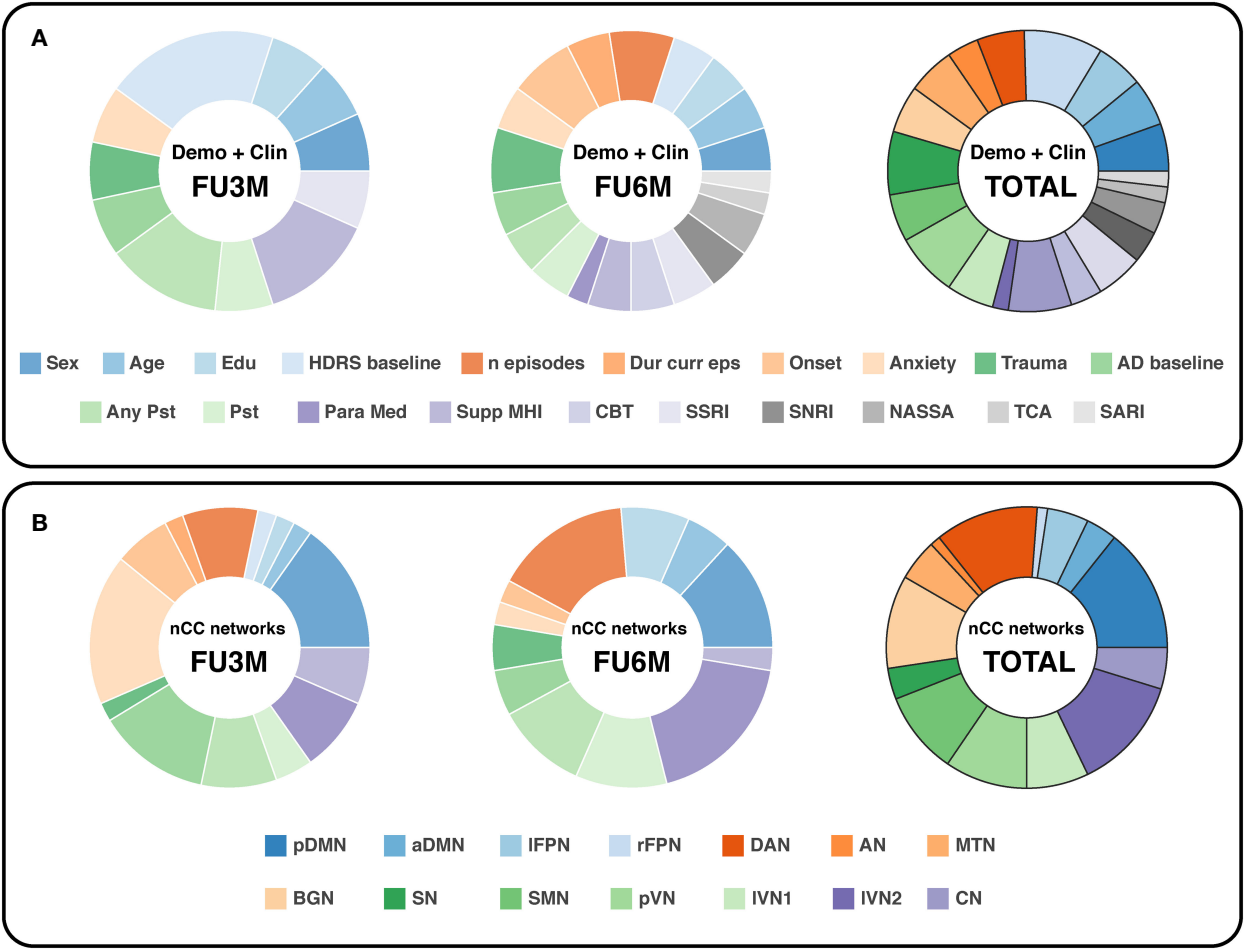
Doughnut charts reflecting the most important demographic/clinical **(A)** and nCC **(B)** predictors. The number of predictors was counted by summing all optimal k predictors (with Kruskal-Wallis feature selection) of the binary and continuous prediction models. demo, demographic, clin, clinical; nCC, number of coherence clusters; FU3M/6M, follow-up at 3 or 6 months; HDRS, Hamilton Depression Rating Scale; Dur curr eps, duration of current episode; AD, antidepressant; Pst, psychotherapy; Para Med, paramedical profession; Supp MHI, support from a mental health institution; CBT, cognitive behavioral therapy; SSRI, selective serotonin reuptake inhibitors; SNRI, serotonin and norepinephrine reuptake inhibitors; NASSA, noradrenaline and specific serotonergic antidepressants; TCA, tricyclic antidepressants; SARI, serotonin antagonist and reuptake inhibitors; pDMN/aDMN, posterior/anterior default mode network; lFPN/rFPN, left/right frontoparietal network; DAN, dorsal attention network; AN, auditory network; MTN, medial temporal network; BGN, basal ganglia network; SN, salience network; SMN, sensorimotor network; pVN, primary visual network; lVN1/lVN2, lateral visual network 1/2; CN, cerebellum network.

For the demographic and clinical features, the HDRS at baseline was slightly the most important feature, see **Figure 6A**. From the regression analysis, the β-coefficient estimate of the independent predictor HDRS baseline was -3.9, indicating that a higher HDRS at baseline in general leads to more improvement, see **Supplementary Table 7**. Remarkably, two treatment-related variables were highly valuable for prediction at 3-months follow-up: support from a mental health institution and having any type of psychotherapy in the month before study participation. On the other hand, more depression history-based predictors were valuable for the 6-months prediction: first onset of depression, number of lifetime episodes and total trauma score. These parameters all had positive β-coefficient estimates, indicating that higher scores lead to an increase in depression severity change, and thus less improvement. It has to be noticed that for the 6-months follow-up predictions, the importance between the features was more equally distributed.

In terms of nCC networks, two networks were encountered consistently in the optimal models for both follow-up predictions: the pDMN and DAN, see **Figure 6B**. The BGN and lVN2 were the most dominant for the 3-months and 6-months follow-up, respectively. Furthermore, the visual networks were relatively abundant in total. Especially the pVN and lVN2 were well represented in the 3- and 6-months predictions.

Second, there was one feature category that performed the most optimal for all prediction methods and was found to be significant after multiple comparison correction in the LOOCV continuous prediction: the nCC model predicting the depression severity change at 3-months follow-up. For both continuous approaches, the model was optimal with the identical k = 5 features. The regression model over the data of all subjects was as follows:


(4) $$\begin{array}{c} \Delta \text{HDR}{\text{S}}_{\text{FU}3\text{M}-\text{baseline}}\\ \;=191-4.51\times \text{nCC}\_\text{SMN}\_\text{pVN}-3.24\\ \;\;\times \text{nCC}\_\text{pDMN}\_\text{lVN}2-2.96\times n\text{CC}\_\text{SMN}\_\text{CN}\\ \;\;-2.64\times \text{nCC}\_\text{pDMN}\_\text{BGN}-2.54\\ \;\;\times \text{nCC}\_\text{MTN}\_\text{BGN} \end{array}$$

With the dependent variable *ΔHDRS_FU3M-baseline_* = HDRS change after 3-months compared to baseline, and the five predictors *nCC* for the SMN-pVN, pDMN-lVN2, SMN-CN, pDMN-BGN and MTN-BGN pair, in order of strongest predictor significance to the model.

**Figure 7:**
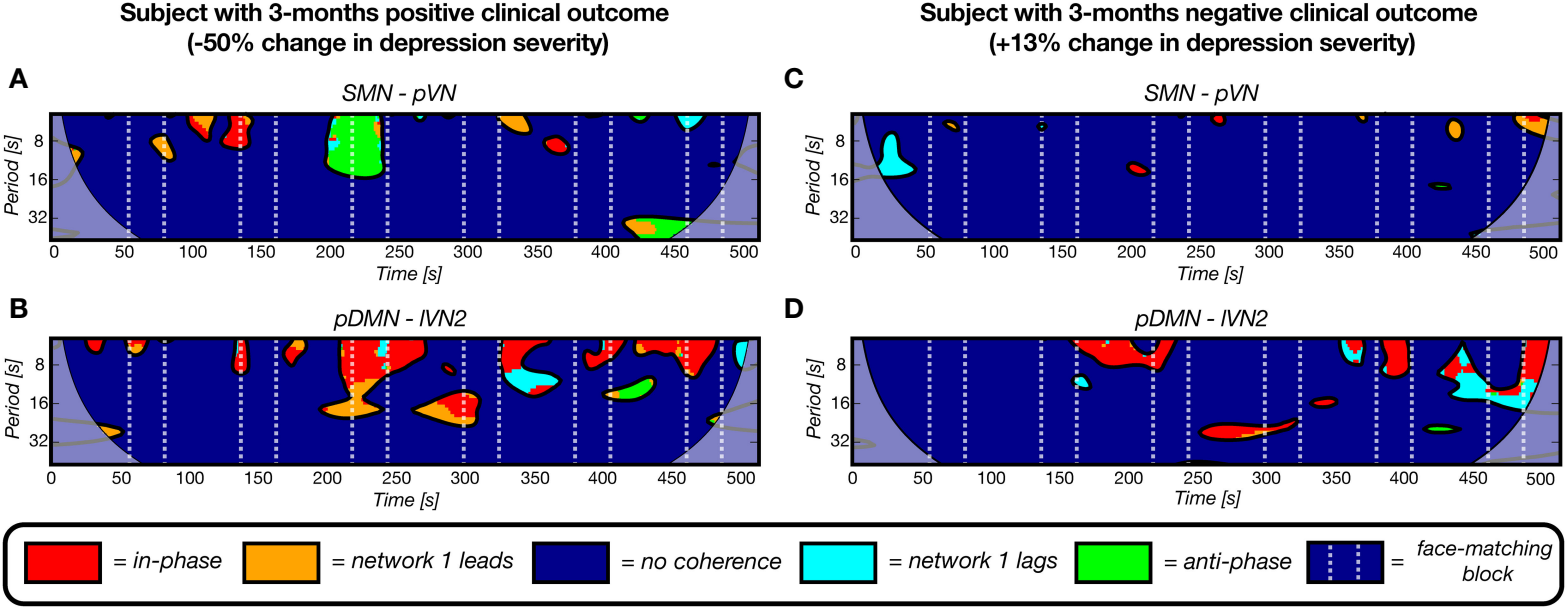
Four representative wavelet coherence maps, derived from fMRI scans at baseline, of two network pairs for two subjects with a positive **(A, B)** and negative **(C, D)** clinical outcome after 3 months (-50% and +12% change in depression severity, respectively). The number of coherence clusters of these two network pairs at baseline were found to be significantly predictive of depression severity change after 3 months in a linear regression model. A) The maps for a subject with a positive clinical outcome show more periods of coherence (clusters) for the sensorimotor network – primal visual network pair and for the posterior default mode network – lateral visual network 2 pair. The maps of a subject with a negative 3-months clinical outcome illustrate the opposite pattern as reflected by the sparsity.

From Equation 4 and **Supplementary Table 8** it can be derived that a higher number of clusters for all five pairs contributes to a decline in HDRS change severity, i.e. more improvement. In **Figure 7**, four examples of wavelet coherence maps of the two most significant network pairs are plotted for two subjects, one with a positive clinical outcome (**Figures 7A, B**) and one with a negative clinical outcome (**Figures 7C, D**). Here, the sparsity in the wavelet coherence map can be clearly observed for the subject with a negative clinical outcome, especially for the SMN-pVN pair. Moreover, the group means ± std for the nCC of the two networks pairs are 15.4 ± 2.26 vs. 13.7 ± 3.37 for the SMN-pVN pair and 14.9 ± 1.73 vs. 11.9 ± 3.79 for the pDMN-lVN2 pair for the positive vs. negative clinical outcome, respectively. For the 6-month follow-up prediction, the same pattern was observed: a higher nCC for 7 out of 8 network pairs (apart from lVN1-lVN2) in the optimal model were associated with a decrease in depression severity, see **Supplementary Table 8**.

## 4 Discussion

In this study, the potential of dynamic fMRI features for the prediction of clinical outcome after 3- and 6-months in depression was evaluated. Activation, static and dynamic features were extracted from an fMRI dataset of an emotional face-matching task and compared separately, as well as with the addition of demographic and clinical parameters. We found that the wavelet coherence based nCC, a measure of the number of interactions (in-phase, lead, lag or anti-phase) between a pair of networks during an fMRI session, achieved the highest prediction performance with 90.6% and 77.4% accuracy for the 3-month and 6-month clinical outcome, respectively. This was demonstrated with a binary classification. Regression models predicting HDRS change on a continuous level further demonstrated the potential value of nCC. The networks that contributed the most were the pDMN, DAN and two visual networks pVN and lVN2. For leadCoh, a measure of dynamic causality between brain networks, addition of demographic and clinical parameters improved the binary prediction at 3-months to 81.2% accuracy.

The fact that nCC outperformed the other categories confirmed our hypothesis that a dynamical approach would be more valuable for the prediction of clinical outcome in depression compared to conventional analysis methods such as task-related brain activity or sFC. Furthermore, an ensemble of leadCoh with demographic and clinical information also resulted in the second highest accuracy achieved in this study. The wavelet coherence maps reveal comprehensive information, such as the type and duration of interaction per frequency bin and per time unit. On the contrary, activation and sFC ignore the temporally complex mechanisms of the brain. For example, brain studies have shown that network interactions are fluctuating in a temporally coordinated organization, both in rest and during tasks (59, 66). Moreover, the time spent in certain states or patterns of network FC has been found to be subject-specific (66). Therefore, including dynamic information into the prediction models, could have enhanced the prediction of depression symptom improvement on an individual level.

While it was hypothesized that aberrant coherence between frontal and limbic networks would predict clinical outcome, it was found that the pDMN, DAN and two visual networks occurred the most frequently and consistently for both follow-ups. Several previous studies confirm the current findings of abnormal dynamic interactions between the DMN, DAN and visual networks in MDD (19–21, 67). However, these were based on dFC during resting-state and compared between MDD and healthy controls. As mentioned earlier, there is one paper that counted the number of coherence clusters using WCA for classification between MDD and healthy controls in resting-state (26). The most important pair that discriminated between MDD and controls was the CN-lateral motor network pair. An increase in the number of clusters was found for this pair in MDD, which they attributed to a disruptive interaction between the cerebellum and control network, potentially explaining lower motor activity in MDD patients. In our study, we also found a similar pair, SMN-CN, that was important for the 3-month follow-up prediction (see Equation 3). The mean nCC of our MDD group was also higher (mean 13.9 ± 3.1) than the controls reported in that study (median around 5). Yet, it is hard to compare as a network pair related to motor control is probably likely to be more often interactive during task periods than in rest. Moreover, the MDD subjects with a positive clinical outcome had a higher nCC (16.8 ± 4.3) than the subjects with a negative clinical outcome (13.0 ± 2.1). Finally, one study conducted WCA to differentiate MDD subjects from healthy controls in resting-state electroencephalography by using the wavelet maps as input to a convolutional neural network. Wavelet coherence between electrodes on DMN regions, especially on top of the posterior located precuneus and lateral parietal cortex, were found to be the most accurate biomarkers and significantly different between groups (68). Similarly, we found the pDMN to be a significant predictor for both follow-ups.

Given that most studies that found abnormal connectivity between frontal and limbic networks were based on resting-state fMRI whereas this study implemented a task paradigm involving attention and sensory processing, the difference in results is not surprising. There is increasing evidence of cognitive deficits in depression, involving attention, concentration and goal-directed behavior (69). The DAN is associated with goal-directed attention (70), i.e. having sustained focus on a specific task while suppressing non-relevant stimuli. Furthermore, the posterior cingulate cortex hub of the pDMN, is involved in emotion and cognition (71). The significant model contribution of nCC between the pDMN, DAN and visual networks in the models of may therefore be generally explained as deficits in sustained attention and visual and affective processing of emotion-related cues. More specifically, a lower number of coherence clusters, reflecting network interactions, was associated with a poorer clinical outcome. This was observed for predicting the 3-month and 6-month depression severity change. Perhaps, the lack of interactions between these networks reflects a diminished capability to remain focused and process the stimuli.

The other dynamic feature category dFC did not appear to have high predictive value. dFC reflects the fluctuation of activation synchronicity between networks. The dFC is proportional to the number and duration of in-phase clusters of the wavelet coherence (the higher the standard deviation of functional connectivity, the more and rapidly-changing in-phase clusters). The fact that the dFC performances was low compared to nCC could be explained by the fact that the nCC also incorporates the lag-phase and anti-phase coherences. Thus, instead of in-phase reductions, perhaps there was a reduction of lag-phase or anti-phase wavelet coherence clusters in subjects with a negative clinical outcome. Interestingly, anti-correlations of the DMN and DAN have been found to be predictive of clinical outcome following transcranial magnetic stimulation therapy in depression (72, 73). This was demonstrated for resting-state and task-based data, even including the Hariri task (73). Patterns of lag and anticorrelation may indicate affected regulatory interactions between networks, such as processes of inhibitory or neurofeedback nature (74).

When comparing the follow-ups, it can be observed that for most of the feature categories the 3-months prediction was higher than the 6-months prediction. Potentially, the fMRI-only features derived from baseline scans provide a more accurate prognosis for the short-term but loses predictive performance for follow-ups at a later stage. This theory is supported by the fact that the most important features for the 6-month predictions include parameters that are related to the history of depression: the age of onset, previous number of episodes and the total trauma score. These parameters were included in the top feature sets for all 6-months demo+clin prediction models. On the contrary, the top demographic and clinical variables for the 3-months prediction are more contemporary variables such as the baseline HDRS, support from a mental health institution or having received any form of psychotherapy in the month prior to the study participation. This further suggests that prediction of short-term severity change relies more on features that are momentary, i.e. only having effect on the depression state of a patient for a limited period, whereas long-term severity change prediction may improve by including more permanent, i.e. history-based, information. Future studies could repeat MRI scans after several months and compare to earlier scans to investigate whether the prediction accuracy improves. Nonetheless, the addition of demo+clin variables did not seem to improve the prediction of the change in depression severity on a continuous level at both follow-ups, except for sFC. Other follow-up studies are required to evaluate the added value of demographic and clinical information for prognosis of depression purposes.

It is noteworthy that the HDRS scale, on which all dependent variables are based, has been criticized as there has been little research on the clinical relevance of changes in relative HDRS scores (75, 76). For example, an absolute reduction of HDRS score of 10 could be clinically different when the participant changes from 35 to 25 or from 15 to 5. Moreover, a decrease in 50% might be obtained more easily with a high baseline score. Subsequently, follow-up studies have been conducted that validated the use of 50% cutoff for relative change in HDRS as measure of clinically significant improvement (60, 75, 76). For validation purposes, they compared the absolute and relative changes in HDRS to the Clinical Global Impressions Scale (77), an instrument often implemented in clinical practice as it is interpreted as intuitive to clinical experts and has good inter-rater reliability (76, 77). Their results demonstrate that absolute change in HDRS, rather than relative change, was influenced by baseline severity and that the 50% threshold was clinically justified. Nonetheless, we assessed the predictive value of the feature categories using absolute change in HDRS as dependent variable. For this, we implemented the continuous LOOCV regression-based method. The results (**Supplementary Table 9**), indicate that the nCC category still reached high performance as well as brain activation. Yet, the demo+clin category scored the highest for both follow-ups. After further inspection, the HDRS at baseline was the top predictor in both scenarios, confirming the findings of these validation studies that absolute changes in HDRS are more biased by baseline HDRS scores than relative changes. Therefore, the analyses of this study were conducted based on the relative change in HDRS with the 50% threshold for the binary classification.

There are a few limitations to be addressed in this study. The unbalanced dataset, especially at 3-months follow-up, caused some bias in the classification performance. This disbalance between two groups tends models to predict the unseen data rather as the majority class. However, in this study, the Kruskall-Wallis feature selection resulted in more balanced sensitivity-specificity ratios. The SVM-RFE feature selection method is a wrapper-based method which fits a model on all the training data points taken together. It has already been shown that the SVM-RFE underperforms compared to simplistic filter-based methods in classification of unbalanced datasets (78). In such cases, the trained model often yields skewed decision boundaries. The Kruskall-Wallis method, however, is a filter-based method which allows testing between unequal sample sizes and does not assume a normal distribution. Potentially, these properties resulted in more balanced performance for the follow-up predictions. Moreover, the imbalance problem was alleviated by applying a higher penalty for classifying the minority class incorrectly. Another limit of the study is the relatively small sample size. We implemented SVM classification with leave-one-out cross-validation, which has been shown to be more optimal on smaller datasets by reducing the potential of overfitting and increasing the amount of training samples (79, 80). Yet, overfitting can still occur (80). By strict feature selection, where the number of features was smaller than the number of subjects, we further endeavored to reduce the risk of overfitting. Nonetheless, follow-up studies with larger sample sizes are required to assess whether the current findings generalize to larger populations. Additionally, the optimal models in this study were not evaluated on an external independent dataset. The optimization of *k* is similar to a grid search where the LOOCV procedure was repeated over a range of *k* from 1-20, and in each iteration its performance was validated over all folds. As the parameter *k* was optimized during training and validation involving all samples of the dataset (there was no sample that was untouched, i.e. not used for training nor validation), overfitting of the optimal models to the study’s dataset might still have occurred. To assess the generalizability and robustness of the predictive nCC models for MDD prognosis, it is necessary to test the optimal models on an external independent dataset. Since the sample size of this study was relatively small, splitting the dataset up into such independent test set would not provide reliable results. This requires a dataset with a larger sample size and similar data acquisition parameters (most importantly, relatively high temporal and spatial resolution). With the recent and continuous improvements in acquisition techniques, such repetition study is feasible.

The study also has several strengths. First, multi-echo and multiband imaging was implemented, which improves the signal-to-noise ratio while minimizing signal loss and still achieving relatively high spatial (2.29 x 2.29 x 2.70 mm3) and temporal (1.35 s) resolution. This enhances the quality of the fMRI data and allows for more reliable temporal and spectral analyses, such as the implemented dFC and WCA. Second, subjects were selected with strict selection criteria, thereby reducing potentially biased effects of comorbidities. Even though MDD is known to be a heterogeneous disease and often presented with comorbidities, it is priority to comprehend the brain mechanisms of separate diseases first. Third, the implementation of the continuous HDRS change prediction further evaluates the predictive value of features more accurately whereas the performance of a binary method could be inflated by uncertain samples concentrated around the decision boundary. Finally, by assessing separate feature models and combination thereof with ensemble models, we provide a comprehensive analysis and improved clinical outcome prediction which in the future may support the clinical decision-making for subjects with depression.

In conclusion, the majority of functional MRI research related to depression prognosis is based on static activity or connectivity measures. In the present study, we analyzed the value of temporally varying interactions between functional brain networks on improving the prediction of change in depression severity on a binary and continuous scale. The number of interactions during an emotional face-matching fMRI session, expressed as the number of coherence clusters derived from a wavelet coherence analysis map, was found to be most predictive of change in depression severity at 3 and 6 months. The pDMN, DAN and two visual networks contributed most to the highest performance. Generally, a lower number of network interactions was predictive of poorer clinical outcome, suggesting that patients who did not improve may have more difficulties with remaining focused on, and processing of emotion-related stimuli. The performance of predicting change in depression severity was higher for the 3-months than the 6-months follow-up. Addition of demographic and clinical variables to the “lead coherence” wavelet feature improved the binary classification of the 3-months prediction for this feature category.

Overall, the paper contributions present as threefold:

1) To the authors’ knowledge, for the first time dynamic WCA clusters have been investigated for depression prognosis purposes.
2) With this feature, a high accuracy of 87.5% and 77.4% was obtained for predicting depression severity changes after 3 and 6 months, respectively.
3) The brain networks that were found to be most important for prediction partially confirm previous findings in literature, mainly the abnormal interactions of the DMN and DAN.

These results reflect the complexity and synergy of the rapidly changing brain states which should be taken into account when analyzing fMRI data in psychiatric disorders. Larger studies are required to assess its generalizability to a larger population.

## Data availability statement

The datasets presented in this article are not readily available because of privacy regulations. However, the processed data from which the results were derived may be provided upon request. Requests to access the datasets should be directed to j.pilmeyer@tue.nl.

## Ethics statement

The studies involving humans were approved by Medical Ethical Review Commission, Maxima Medical Centre, Veldhoven, the Netherlands (W20.054). The studies were conducted in accordance with the local legislation and institutional requirements. The participants provided their written informed consent to participate in this study.

## Author contributions

JP: Writing – review & editing, Writing – original draft, Visualization, Validation, Software, Methodology, Investigation, Formal Analysis, Data curation, Conceptualization. RL: Writing – review & editing, Supervision, Software, Resources, Methodology, Conceptualization. FR: Writing – review & editing, Methodology, Investigation. JJ: Writing – review & editing, Validation, Supervision, Methodology, Conceptualization. MB: Writing – review & editing, Validation, Supervision, Methodology, Conceptualization. SZ: Writing – review & editing, Supervision, Project administration, Methodology, Conceptualization.
