# Supplementary material for: Improved clinical outcome prediction in depression using neurodynamics in an emotional face-matching functional MRI task
Source: Front Psychiatry. 2024 Mar 22;15:1255370. doi: 10.3389/fpsyt.2024.1255370 (PMC10996064; doi:10.3389/fpsyt.2024.1255370)
Supplement: Supplementary file 1 [file DataSheet_1.docx]

Supplementary Material

Improved prediction of the course of depression using neurodynamics in an emotional face-matching functional MRI task

**Jesper Pilmeyer^*^, Rolf Lamerichs, Faroeq Ramsaransing, Jacobus F.A. Jansen, Marcel Breeuwer, Svitlana Zinger**

*** Correspondence:** Jesper Pilmeyer: [j.pilmeyer@tue.nl](mailto:j.pilmeyer@tue.nl)

# Supplementary Figures and Tables

**
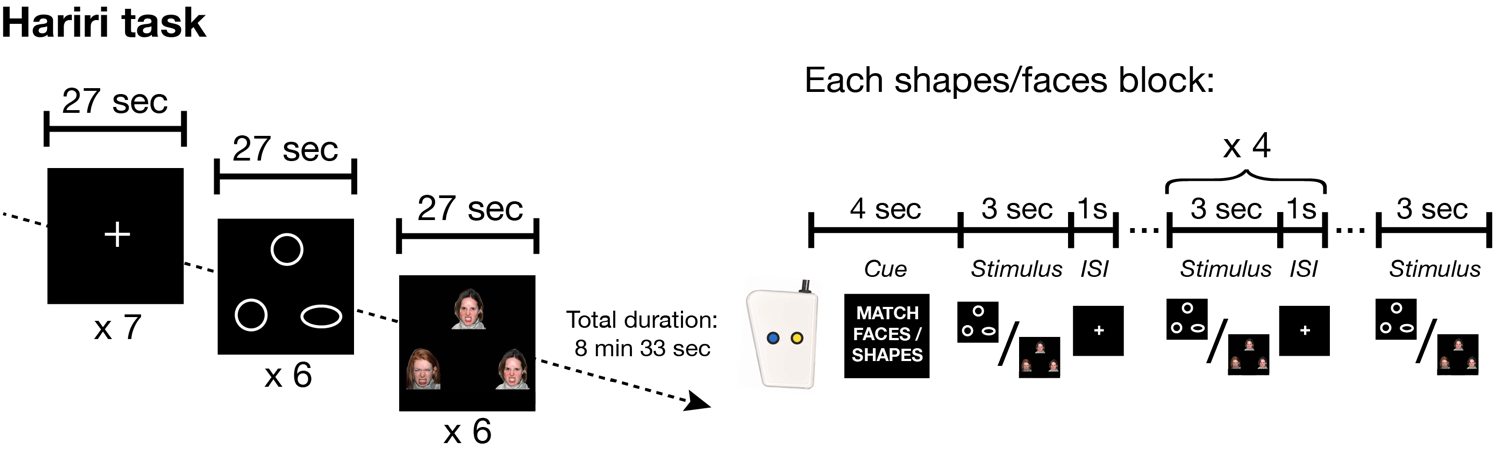
**

**Supplementary Figure 1** The emotion face-matching Hariri task contains 7 blocks of rest (cross), 6 periods of matching shapes and 6 period of matching negative emotions (anger and fear). Each block lasts 27 seconds and contains a cue (4 sec) with instructions, 6 stimuli (3 sec each) and an inter-stimulus interval (ISI) of 1 sec between each stimulus. Participants are instructed to use the button press (shown in the image left to the Cue block) to match the left or right lower image to the upper image during the shapes and faces blocks.

**Supplementary Table 1** Performance of the all classifiers for the binary 3- and 6-month clinical outcome prediction. Abbreviations: demo = demographic, clin = clinical; Act = activity; s/dFC = static/dynamic functional connectivity; leadCoh = lead coherence; nCC = number of coherence clusters; SVM-RFE = support vector machine – recursive feature elimination; acc = accuracy; sens = sensitivity; spec = specificity; prec = precision; AUC = area under the curve.

|  |  |  | **3-months follow-up** | | | | | | | |
| --- | --- | --- | --- | --- | --- | --- | --- | --- | --- | --- |
| **Feature category** | | | **Feature selection** | **Optimal k** | **Acc** | **Sens** | **Spec** | **Prec** | **F1-score** | **AUC** |
| **Demo/clin** | | | SVM-RFE | 1 | 71.9 | 0.00 | 95.8 | 0.000 | NaN | 0.479 |
| **Act** | | | SVM-RFE | 17 | 62.5 | 37.5 | 70.8 | 0.300 | 0.333 | 0.542 |
| **sFC** | | | SVM-RFE | 15 | 78.1 | 37.5 | 91.7 | 0.600 | 0.462 | 0.646 |
| **dFC** | | | SVM-RFE | 16 | 62.5 | 25.0 | 75.0 | 0.250 | 0.250 | 0.500 |
| **leadCoh** | | | SVM-RFE | 13 | 65.6 | 37.5 | 75.0 | 0.333 | 0.353 | 0.563 |
| **nCC** | | | SVM-RFE | 20 | **90.6** | **62.5** | **100** | **1.00** | **0.769** | **0.813** |
| **Demo/clin** | | | Kruskal-Wallis | 10 | 68.8 | **62.5** | 70.8 | 0.417 | 0.500 | 0.667 |
| **Act** | | | Kruskal-Wallis | 2 | 59.4 | 37.5 | 66.7 | 0.273 | 0.316 | 0.521 |
| **sFC** | | | Kruskal-Wallis | 15 | 68.8 | 25.0 | 83.3 | 0.333 | 0.286 | 0.542 |
| **dFC** | | | Kruskal-Wallis | 20 | 65.6 | 50.0 | 70.8 | 0.364 | 0.421 | 0.604 |
| **leadCoh** | | | Kruskal-Wallis | 10 | 75.0 | 50.0 | 83.3 | 0.500 | 0.500 | 0.667 |
| **nCC** | | | Kruskal-Wallis | 13 | 87.5 | **62.5** | 95.8 | 0.833 | 0.714 | 0.792 |

|  |  | **6-months follow-up** | | | | | | | |
| --- | --- | --- | --- | --- | --- | --- | --- | --- | --- |
| **Feature category** | | **Feature selection** | **Optimal k** | **Acc** | **Sens** | **Spec** | **Prec** | **F1-score** | **AUC** |
| **Demo/clin** | | SVM-RFE | 5 | 64.5 | 41.7 | 78.9 | 0.556 | 0.476 | 0.603 |
| **Act** | | SVM-RFE | 2 | 77.4 | 66.7 | 84.2 | 0.727 | 0.696 | 0.754 |
| **sFC** | | SVM-RFE | 2 | 41.9 | 25.0 | 52.6 | 0.250 | 0.250 | 0.388 |
| **dFC** | | SVM-RFE | 14 | 64.5 | 50.0 | 73.7 | 0.545 | 0.522 | 0.618 |
| **leadCoh** | | SVM-RFE | 5 | 64.5 | 50.0 | 73.7 | 0.545 | 0.522 | 0.618 |
| **nCC** | | SVM-RFE | 8 | 77.4 | 66.7 | 84.2 | 0.727 | 0.696 | 0.754 |
| **Demo/clin** | | Kruskal-Wallis | 15 | 67.7 | 66.7 | 68.4 | 0.571 | 0.615 | 0.675 |
| **Act** | | Kruskal-Wallis | 10 | 67.7 | 75.0 | 63.2 | 0.563 | 0.643 | 0.691 |
| **sFC** | | Kruskal-Wallis | 1 | 64.5 | 58.3 | 68.4 | 0.538 | 0.560 | 0.634 |
| **dFC** | | Kruskal-Wallis | 2 | 67.7 | 66.7 | 68.4 | 0.571 | 0.615 | 0.675 |
| **leadCoh** | | Kruskal-Wallis | 3 | 61.3 | 41.7 | 73.7 | 0.500 | 0.455 | 0.577 |
| **nCC** | | Kruskal-Wallis | 8 | 77.4 | 75.0 | 78.9 | 0.692 | 0.720 | 0.770 |

**Supplementary Table 2** Comparison between predicted and actual depression severity changes for the multiple linear regression with leave-one-out cross-validation approach. Models were fit on the N-1 training set, which was then used to predict depression severity change after 3 and 6 months of the test subject. This procedure was repeated for all subjects. Correlation, RMSE and MAE indicate the performance between predicted and actual changes in severity. The p-value corresponds to the correlation between both. Bold p-values marked with * are significant after multiple comparison correction at p < 0.05. The optimal k features elements of each category were determined by initial support vector machine – recursive feature elimination selection (K = 20) and subsequently removing the lowest significant predictors iteratively until maximum performance was reached. Abbreviations: demo = demographic, clin = clinical; Act = activity; s/dFC = static/dynamic functional connectivity; leadCoh = lead coherence; nCC = number of coherence clusters; RMSE = root mean square error; MAE = mean absolute error.

| **3-months follow-up** | | | | | |
| --- | --- | --- | --- | --- | --- |
| **Feature category** | **Correlation** | **RMSE** | **MAE** | **Optimal k** | **p-value (uncorr.)** |
| **Demo/clin** | **0.489** | **26.9** | **21.9** | 3 | < 0.01 |
| **Act** | 0.256 | 29.4 | 23.8 | 1 | 0.157 |
| **sFC** | -0.062 | 50.1 | 40.8 | 20 | - |
| **dFC** | 0.157 | 40.7 | 31.8 | 15 | 0.391 |
| **leadCoh** | 0.234 | 36.3 | 29.0 | 5 | 0.198 |
| **nCC** | 0.284 | 41.6 | 33.7 | 13 | 0.115 |
| **6-months follow-up** | | | | | |
| **Feature category** | **Correlation** | **RMSE** | **MAE** | **Optimal k** | **p-value (uncorr.)** |
| **Demo/clin** | 0.293 | 38.2 | 30.0 | 20 | 0.110 |
| **Act** | 0.134 | 31.9 | 24.3 | 3 | 0.471 |
| **sFC** | 0.064 | 30.6 | 25.1 | 1 | 0.730 |
| **dFC** | -0.018 | 33.0 | 29.5 | 3 | - |
| **leadCoh** | 0.173 | 27.3 | 22.1 | 2 | 0.352 |
| **nCC** | **0.538** | **25.0** | **20.0** | 5 | **< 0.01 ^*^** |

**Supplementary Table 3** Goodness-of-fit statistics for the optimal multiple linear regression models for each of the feature categories relating to the change in 3- and 6-month depression severity. The models were fit over all subjects and the optimal k features elements were determined by initial support vector machine – recursive feature elimination feature selection (K = 20) and by subsequently removing the lowest significant predictors iteratively until maximum performance was reached. Abbreviations: demo = demographic, clin = clinical; Act = activity; s/dFC = static/dynamic functional connectivity; leadCoh = lead coherence; nCC = number of coherence clusters; RMSE = root mean square error; Adj. R^2^ = adjusted coefficient of determination.

| **3-months follow-up** | | | | |
| --- | --- | --- | --- | --- |
| **Feature category** | **F-statistic** | **RMSE** | **Adj. R^2^** | **Optimal k** |
| **Demo/clin** | 7.74 | 23.6 | 0.395 | 3 |
| **Act** | 4.31 | 24.5 | 0.348 | 5 |
| **sFC** | 6.12 | 21.5 | 0.498 | 6 |
| **dFC** | 5.43 | 23.2 | 0.417 | 5 |
| **leadCoh** | **8.53** | **20.4** | **0.549** | 5 |
| **nCC** | 6.15 | 20.6 | 0.538 | 7 |
| **6-months follow-up** | | | | |
| **Feature category** | **F-statistic** | **RMSE** | **Adj. R^2^** | **Optimal k** |
| **Demo/clin** | 4.38 | 20.0 | 0.474 | 8 |
| **Act** | 6.96 | 20.6 | 0.443 | 4 |
| **sFC** | 5.59 | 22.9 | 0.314 | 3 |
| **dFC** | **7.66** | 19.0 | 0.536 | 5 |
| **leadCoh** | 4.55 | 20.4 | 0.453 | 7 |
| **nCC** | 6.58 | **16.9** | **0.626** | 9 |

**Supplementary Table 4** Performance of the ensemble classifiers for the binary 3- and 6-month clinical outcome prediction with SVM-RFE (support vector machine – recursive feature elimination) future ranking. Abbreviations: demo = demographic, clin = clinical; Act = activity; nCC = number of coherence clusters; s/dFC = static/dynamic functional connectivity; leadCoh = lead coherence.

|  | **3-months follow-up** | | | | | | |
| --- | --- | --- | --- | --- | --- | --- | --- |
| Ensemble | **Feature selection** | **Acc** | **Sens** | **Spec** | **Prec** | **F1-score** | **AUC** |
| **Demo/clin + Act** | SVM-RFE | 62.5 | 12.5 | 79.2 | 0.167 | 0.143 | 0.458 |
| **Demo/clin + sFC** | SVM-RFE | 68.8 | 0 | 91.7 | 0 | NaN | 0.458 |
| **Demo/clin + dFC** | SVM-RFE | 62.5 | 0 | 83.3 | 0 | NaN | 0.417 |
| **Demo/clin + leadCoh** | SVM-RFE | 68.8 | 12.5 | 87.5 | 0.250 | 0.167 | 0.500 |
| **Demo/clin + nCC** | SVM-RFE | **78.1** | **25.0** | **95.8** | **0.667** | **0.364** | **0.604** |
|  | **6-months follow-up** | | | | | | |
| Ensemble | **Feature selection** | **Acc** | **Sens** | **Spec** | **Prec** | **F1-score** | **AUC** |
| **Demo/clin + Act** | SVM-RFE | 48.4 | 16.7 | 68.4 | 0.250 | 0.200 | 0.425 |
| **Demo/clin + sFC** | SVM-RFE | 32.3 | 8.33 | 47.4 | 0.091 | 0.087 | 0.279 |
| **Demo/clin + dFC** | SVM-RFE | 51.6 | 8.33 | **79.0** | 0.200 | 0.118 | 0.436 |
| **Demo/clin + leadCoh** | SVM-RFE | **58.1** | **41.7** | 68.4 | **0.455** | **0.435** | **0.550** |
| **Demo/clin + nCC** | SVM-RFE | 48.4 | 25.0 | 63.2 | 0.300 | 0.273 | 0.441 |

**Supplementary Table 5** Performance of the ensemble classifiers for the binary 3- and 6-month clinical outcome prediction with Kruskal-Wallis future ranking. Abbreviations: demo = demographic, clin = clinical; Act = activity; nCC = number of coherence clusters; s/dFC = static/dynamic functional connectivity; leadCoh = lead coherence.

|  | **3-months follow-up** | | | | | | |
| --- | --- | --- | --- | --- | --- | --- | --- |
| Ensemble | **Feature selection** | **Acc** | **Sens** | **Spec** | **Prec** | **F1-score** | **AUC** |
| **Demo/clin + Act** | Kruskal-Wallis | 71.9 | 50.0 | 79.2 | 0.444 | 0.471 | 0.646 |
| **Demo/clin + sFC** | Kruskal-Wallis | 78.1 | 50.0 | 87.5 | 0.571 | 0.533 | 0.688 |
| **Demo/clin + dFC** | Kruskal-Wallis | 71.9 | **62.5** | 75.0 | 0.455 | 0.526 | 0.688 |
| **Demo/clin + leadCoh** | Kruskal-Wallis | 81.2 | **62.5** | 87.5 | 0.625 | 0.625 | 0.750 |
| **Demo/clin + nCC** | Kruskal-Wallis | **87.5** | **62.5** | **95.8** | **0.833** | **0.714** | **0.792** |
|  | **6-months follow-up** | | | | | | |
| Ensemble | **Feature selection** | **Acc** | **Sens** | **Spec** | **Prec** | **F1-score** | **AUC** |
| **Demo/clin + Act** | Kruskal-Wallis | 48.4 | 41.7 | 52.6 | 0.357 | 0.385 | 0.472 |
| **Demo/clin + sFC** | Kruskal-Wallis | 45.2 | 25.0 | 57.9 | 0.273 | 0.261 | 0.415 |
| **Demo/clin + dFC** | Kruskal-Wallis | 45.2 | 33.3 | 52.6 | 0.308 | 0.320 | 0.430 |
| **Demo/clin + leadCoh** | Kruskal-Wallis | 58.1 | 41.7 | **68.4** | 0.455 | 0.435 | 0.550 |
| **Demo/clin + nCC** | Kruskal-Wallis | **61.3** | **50.0** | **68.4** | **0.500** | **0.500** | **0.592** |

**Supplementary Table 6** Linear regression performance of ensemble models between fMRI and demographic/ clinical features using support vector machine – recursive feature elimination feature selection. The metrics in this table are a comparison between predicted and actual depression severity changes using a leave-one-out cross-validation approach. Models were fit on the N-1 training set, which was then used to predict depression severity change after 3 and 6 months of the test subject. This procedure was repeated for all subjects. Correlation, RMSE and MAE indicate the performance between predicted and actual changes in severity. The p-value corresponds to the correlation between both. The ensemble predicted severity change was calculated as average between the separate demo/clin model and the separate fMRI-based models. Abbreviations: demo = demographic, clin = clinical; Act = activity; nCC = number of coherence clusters; s/dFC = static/dynamic functional connectivity; leadCoh = lead coherence; RMSE = root mean square error; MAE = mean absolute error.

|  | **3-months follow-up** | | | |
| --- | --- | --- | --- | --- |
| **Ensemble** | **Correlation** | **RMSE** | **MAE** | **p-value (uncorr.)** |
| **Demo/clin + Act** | 0.233 | 40.7 | 30.9 | 0.200 |
| **Demo/clin + sFC** | 0.182 | 38.2 | 32.4 | 0.320 |
| **Demo/clin + dFC** | 0.264 | 34.9 | 30.1 | 0.144 |
| **Demo/clin + leadCoh** | 0.327 | **34.5** | 29.7 | 0.0676 |
| **Demo/clin + nCC** | **0.374** | 35.0 | **27.9** | **0.0351** |
|  | ***6-months follow-up*** | | | |
| **Ensemble** | **Correlation** | **RMSE** | **MAE** | **p-value (uncorr.)** |
| **Demo/clin + Act** | 0.195 | 40.5 | 33.1 | 0.292 |
| **Demo/clin + sFC** | -0.0380 | 42.2 | 32.9 | 0.839 |
| **Demo/clin + dFC** | 0.1456 | 36.7 | 29.5 | 0.434 |
| **Demo/clin + leadCoh** | 0.0863 | 46.0 | 33.5 | 0.644 |
| **Demo/clin + nCC** | **0.438** | **29.1** | **22.3** | **0.0137** |

**Supplementary Table 7** The optimal multiple linear regression model for the demographic and clinical features, fit over the data of all subjects and using Kruskall-Wallis feature selection, for each of the feature categories predicting the change in 3- and 6-month depression severity. Abbreviations: HDRS = Hamilton Depression Rating Scale; Pst = psychotherapy; Supp MHI = support from a mental health institution; CBT = cognitive behavioral therapy; NASSA = noradrenaline and specific serotonergic antidepressants; Dur curr eps = duration of current episode; SE = standard error; t-stat = t-statistic.

|  | **3-months follow-up** | | | |  |
| --- | --- | --- | --- | --- | --- |
| **Predictors** | **Estimate** | **SE** | **t-stat** | **p-value** |  |
| **Intercept** | 44.6 | 23.5 | 1.90 | 0.0681 |  |
| **HDRS baseline** | -3.92 | 0.975 | -4.02 | < 0.001 |  |
| **Any Pst** | 42.2 | 11.2 | 3.78 | < 0.001 |  |
| **Supp MHI** | -18.6 | 9.86 | -1.89 | 0.0691 |  |
|  | ***6-months follow-up*** | | | | |
| **Predictors** | **Estimate** | **SE** | **t-stat** | **p-value** |  |
| **Intercept** | -211.7 | 49.9 | -4.25 | < 0.001 |  |
| **CBT** | 69.4 | 16.3 | 4.26 | < 0.001 |  |
| **Onset** | 0.997 | 0.317 | 3.15 | < 0.01 |  |
| **n episodes** | 24.6 | 7.82 | 3.14 | < 0.01 |  |
| **NASSA** | -33.7 | 14.1 | -2.40 | < 0.05 |  |
| **Trauma** | 1.07 | 0.489 | 2.19 | < 0.05 |  |
| **Sex** | 19.0 | 9.19 | 2.07 | 0.0506 |  |
| **Anxiety** | 1.50 | 0.783 | 1.91 | 0.0691 |  |
| **Dur curr eps** | 1.04 | 0.645 | 1.61 | 0.122 |  |

**Supplementary Table 8** The optimal multiple linear regression mode for the nCC feature, fit over the data of all subjects and using Kruskall-Wallis feature selection, for each of the feature categories predicting the change in 3- and 6-month depression severity. Abbreviations: nCC = number of coherence clusters; pDMN = posterior default mode network; DAN = dorsal attention network; MTN = medial temporal network; BGN = basal ganglia network; SMN = sensorimotor network; pVN = primary visual network; lVN1/lVN2 = lateral visual network 1/2; CN = cerebellum network.

|  | **3-months follow-up** | | | |  |
| --- | --- | --- | --- | --- | --- |
| **Predictors** | **Estimate** | **SE** | **t-stat** | **p-value** |  |
| **Intercept** | 191.0 | 36.8 | 5.19 | < 0.001 |  |
| **nCC SMN-pVN** | -4.51 | 1.20 | -3.74 | < 0.001 |  |
| **nCC pDMN-lVN2** | -3.24 | 1.06 | -3.06 | < 0.01 |  |
| **nCC SMN-CN** | -2.96 | 1.22 | -2.43 | < 0.05 |  |
| **nCC pDMN-BGN** | -2.64 | 1.21 | -2.18 | < 0.05 |  |
| **nCC MTN-BGN** | -2.54 | 1.20 | -2.12 | < 0.05 |  |
|  | ***6-months follow-up*** | | | | |
| **Predictors** | **Estimate** | **SE** | **t-stat** | **p-value** |  |
| **Intercept** | 176.2 | 35.5 | 4.96 | < 0.001 |  |
| **nCC lVN1-lVN2** | 4.02 | 0.994 | 4.04 | < 0.001 |  |
| **nCC pDMN-lVN2** | -3.26 | 1.01 | -3.22 | < 0.01 |  |
| **nCC DAN-lVN1** | -3.63 | 1.26 | -2.88 | < 0.01 |  |
| **nCC DAN-lVN2** | -3.52 | 1.25 | -2.81 | < 0.05 |  |
| **nCC MTN-BGN** | -2.71 | 0.988 | -2.74 | < 0.05 |  |
| **nCC pVN-lVN2** | -2.18 | 1.04 | -2.10 | < 0.05 |  |
| **nCC pDMN-pVN** | -1.85 | 1.08 | -1.71 | 0.101 |  |
| **nCC SMN-lVN1** | -2.11 | 1.28 | -1.65 | 0.113 |  |

|  | **3-months follow-up** | | | | |
| --- | --- | --- | --- | --- | --- |
| **Feature category** | **Correlation** | **RMSE** | **MAE** | **Optimal k** | **p-value (uncorr.)** |
| **Demo/clin** | **0.605** | **12.4** | **10.2** | 2 | < 0.001 |
| **Act** | 0.358 | 14.5 | 11.4 | 1 | < 0.05 |
| **sFC** | 0.065 | 21.2 | 17.0 | 6 | 0.725 |
| **dFC** | 0.052 | 18.1 | 15.4 | 5 | 0.778 |
| **leadCoh** | -0.142 | 31.0 | 23.1 | 9 | - |
| **nCC** | 0.398 | 14.3 | 11.7 | 1 | < 0.05 |
|  | ***6-months follow-up*** | | | | |
| **Feature category** | **Correlation** | **RMSE** | **MAE** | **Optimal k** | **p-value (uncorr.)** |
| **Demo/clin** | **0.493** | 14.3 | **10.4** | 7 | < 0.01 |
| **Act** | 0.458 | **13.2** | 11.3 | 1 | < 0.01 |
| **sFC** | 0.072 | 28.9 | 24.1 | 18 | 0.697 |
| **dFC** | 0.143 | 15.1 | 11.7 | 1 | 0.434 |
| **leadCoh** | -0.152 | 21.6 | 17.7 | 2 | - |
| **nCC** | 0.373 | 18.5 | 15.3 | 13 | < 0.05 |

**Supplementary Table 9** Comparison between predicted and actual depression severity **absolute** changes for the multiple linear regression with leave-one-out cross-validation approach. Models were fit on the N-1 training set, which was then used to predict depression severity change after 3 and 6 months of the test subject. This procedure was repeated for all subjects. Correlation, RMSE and MAE indicate the performance between predicted and actual changes in severity. The p-value corresponds to the correlation between both The optimal k features elements of each category were determined by initial Kruskall-Wallis selection (K = 20) and subsequently removing the lowest significant predictors iteratively until maximum performance was reached. Abbreviations: demo = demographic, clin = clinical; Act = activity; s/dFC = static/dynamic functional connectivity; leadCoh = lead coherence; nCC = number of coherence clusters; RMSE = root mean square error; MAE = mean absolute error.
